# Substratum interactions determine immune response to allogeneic transplants of endothelial cells

**DOI:** 10.3389/fimmu.2022.946794

**Published:** 2022-08-08

**Authors:** Elise C. Wilcox, Elazer R. Edelman

**Affiliations:** ^1^ Institute for Medical Engineering & Science, Massachusetts Institute of Technology, Cambridge, MA, United States; ^2^ Cardiovascular Division, Department of Medicine, Brigham and Women’s Hospital, Harvard Medical School, Boston, MA, United States

**Keywords:** collagen scaffold, antigen presentation, T cell, cytotoxicity, immunomodulation, co-stimulatory molecules, cytokine stimulation

## Abstract

Endothelial cells (ECs) are central to vascular health but also interact with and regulate the immune system. Changes in endothelial state enable immune cells to migrate into the tissue to facilitate repair and fight infection. ECs modulate the function of immune cells through the expression of adhesion molecules, chemokines, major histocompatibility complex (MHC), and an array of co-stimulatory and inhibitor molecules. These interactions allow ECs to act as antigen presenting cells (APCs) and influence the outcome of immune recognition. This study elucidates how EC microenvironment, vascular cell biology, and immune response are not only connected but interdependent. More specifically, we explored how cell-substratum interactions influence EC antigen presentation and co-stimulation, and how these differences affect allorecognition in animal models of cell transplantation. Investigation of EC state was carried out using RNA sequencing while assessment of the allogeneic response includes measurements of immune cell cytotoxic ability, T cell proliferation, cytokine release, serum antibodies, and histological staining. Differences in substratum led to divergent EC phenotypes which in turn influenced immune response to transplanted cells, both due to the physical barrier of matrix-adhesion and differences in expression of surface markers. ECs grown in 2D on tissue culture plastic or in 3D on collagen scaffolds had significantly different basal levels of MHC expression, co-stimulatory and adhesion molecules. When treated with cytokines to mimic an inflammatory state, ECs did not converge to a single phenotype but rather responded differently based on their substratum. Generally, 3D ECs were more responsive to inflammatory stimuli than 2D ECs. These unique expression patterns measured *in vitro* also influence immune recognition *in vivo*. ECs grown in 2D were more likely to provoke a cytotoxic response while 3D ECs induced T cell proliferation. ECs are uniquely configured to sense not only local flow and mechanical forces but a range of markers related to systemic state, including immune function. ECs interact with immune cells with differing results depending on the environment in which the EC-lymphocyte interaction occurs. Therefore, understanding this relationship is essential to predicting and modifying the outcome of EC-immune interacts. We specifically examined the relationship between EC substratum and allorecognition.

## 1 Introduction

Studies of endothelial cells have classically focused on their pluripotent physiology which allows them respond to surface, flow and mechanical effects and regulate the local milieu through dynamic local signaling. However, greater attention is now being to be paid to their role in immunobiology.

Our current understanding of immunobiology, including acquired immunity and tolerance, was built on foundational studies in transplant rejection. Despite leaps and bounds in the field of immunology in the past century, predicting the immune response to transplantation remains challenging. Although, organ transplants are widely used in clinical practice, it is difficult to control cell state within the organ. Biomaterials can be used to provide controlled microenvironments which allows them to be used as a platform to study and modulate the immune response. With this in mind, matrix-embedded cells offer the ideal model system in which to study endothelial immunobiology in the context of transplantation.

Given their location at the interface of the blood stream, ECs are often the first cells to detect circulating cytokines, thus facilitating communication between smooth muscle cells, dendritic cells (DCs), macrophages, and T cells. Through their interactions with immune cells and other professional antigen presenting cells (APCs), ECs participate in antigen presentation and immunomodulation. The specific array of co-stimulatory molecules expressed by ECs is determined by their microenvironment, which can determine whether immune cell interactions promote tolerance or rejection. Differences also exist between ECs of different vascular beds as EC function is controlled by the microenvironment (1, 2). This sensitivity makes it challenging to investigate the true relationship between EC phenotype and immune response when ECs are removed from their *in vivo* environment and cultured *in vitro*.

Destruction of the endothelium can be seen in the chronic rejection pathologies of a number of solid organ transplants including of the heart, liver and kidneys (3). ECs constitutively present MHC class I and can be induced to express MHC class II as well as a range of co-stimulatory and inhibitory molecules that are exclusively expressed by immune cells (3). For example, *in vitro*, ECs can stimulate resting T cells while other parenchymal cells such as fibroblasts, epithelial cells, and smooth muscle cells cannot (3). The interaction of ECs with CD4+ T cells through MHC class II signaling has been implicated in graft rejection (4). Presentation of processed self-antigen on MHC class II (and through cross-presentation on MHC class I) has been suggested to be involved in the induction and maintenance of T cell tolerance, indicating an EC-specific role in promoting transplant tolerance as well (5).

One unique aspect of ECs is that they are sided cells – sensing blood flow from above, neighboring cells from their side and mechanical forces from below. The substratum on which ECs reside plays a potent role in regulating all of these senses and define the EC mechanical and surface microenvironment. We focus on how EC-immune cell interactions are affected by substratum and inflammatory signaling. These interactions were investigated using tissue engineered constructs in a model of allogeneic transplantation.

## 2 Materials and methods

### 2.1 Cell culture, cell sources and seeding scaffolds

For initial RNAseq analysis, Sprague-Dawley (SD) Rat Aortic Endothelial Cells (Sigma, R304-05A) were cultured in Rat EC Media (Sigma, R211-500). RNAseq for basal and cytokine conditions as well as for all *in vitro* and *in vivo* assay used Brown Norway (BN) Rat Primary Aortic Endothelial Cells (Cell Biologics, RA-6052BN) cultured in Rat EC Media (Sigma, R211-500). Cytokine treated cells were incubated with media supplemented with 40 ng/ml IFNγ (R&D systems, 585-IF-100) and 25 ng/ml TNFα (R&D systems, 510-RT-010) for 48 hours before isolating RNA. All cells were used between P4-8.

Surgifoam (SF) Absorbable Gelatin Sponges (2mm, Medline, ETH1975) were cut into 0.5 x 0.5 x 0.5 cm^3^ cubes and placed individually in 96-well, flat bottom plates. Scaffolds were hydrated in 200 µl media for at least 1 hour before seeding. For seeding, 5x10^4^ cells in 200 µl were added directly to each scaffold. Seeded scaffolds were cultured overnight undisturbed and then moved to a new plate the next day.

All 2D ECs were confluent before they were treated with cytokines, lysed for isolation of RNA, or lifted for implantation into rats. While determination of cultured EC growth and confluence in 2D can be assessed directly without removal from their growth environment under routine light microscopy, this is not possible for the 3D ECs since they grow both on the surface and inside the porous scaffold. A growth curve, RNAseq analysis, and histology determined that 10-14 days after seeding, 3D ECs are no longer proliferating, are more similar to 2D confluent ECs than 2D subconfluent ECs in terms of their gene expression, and line the inner and outer structures of the scaffold. To more accurately compare confluent 2D ECs sand 3D ECs, all 3D ECs were grown on the scaffold for at least 10 days before isolation of RNA or implantation into rats to ensure they were “confluent” and no longer in a subconfluent, actively proliferating phrase.

### 2.2 Isolation of RNA

Isolation was performed using Qiagen Rneasy Mini Kit (74104), QIAshredder (79654), and RNAse-Free DNAse Set (79254). Cells were rinsed 2x with PBS and disrupted with 350 µl buffer RLT with 10 μl β-ME (2-Mercaptoethanol) per 1 ml Buffer RLT added. The lysate was collected with a rubber policeman and added to the QIAshredder. For the scaffolds, up to 5 scaffolds were added directly to the QIAshredder after rinsing with PBS and disrupted with RLT buffer with β-ME. The remainder of the isolation was carried out following kit instructions, including an on column DNAse digestion step. Three biological replicates were collected for each sample.

### 2.3 RNAseq analysis

Library preparation and sequencing was completed by Novogene using the Illumina Platform. FASTQ files from Novogene were aligned to the Rattus Norvegicus genome from ensembl.org using HISAT2. Aligned reads were counted using featureCounts. Differential gene expression analysis was carried out using DESeq2 (6). Statistically significant genes (p-adj < 0.05, adjusted for multiple hypothesis testing with the Benjamini-Hochberg procedure) with a log2 fold change in expression >1 (upregulation) or <1 (downregulation) were uploaded to g:Profiler tool for functional enrichment analysis (7). Heatmaps of individual genes were created for enriched classes of genes from the Gene Ontology database, primarily gene sets associated with Biological Processes. Heatmaps were scaled using z-scores. Genes with zero or near zero expression across multiple experimental groups were omitted from heatmaps.

### 2.4 Growing ECs on scaffolds and animal surgeries

All animal use was approved by the MIT Institutional Animal Care and Use Committee. There were four groups in each animal experiment: 2D freed (2Df), 3D scaffolded (3DSF), 3D freed (3Df), and sham surgery. For the 3D SF group, ECs were cultured on scaffolds for 14 days before implantation. For both the freed groups, on the day of the implant, cells were trypsined off either 2D tissue culture polystyrene (TCP) or off 3D SF scaffolds (also cultured 14 days). Freed cells were counted and 1x10^6^ ECs were implanted in 50 µl PBS. Freed cells were stored on ice and subcutaneously (SC) implanted in animals between 90-120 minutes after trypsinization. All cell lines were tested for pathogens at the MIT Diagnostic Laboratory of the Division of Comparative Medicine before implantation. Lastly, as a control group, sham surgery rats had an incision and SC pocket made, as with the implant groups, but not receive injections or SF implants.

Transplants were carried out using Brown Norway rat cells (MHC: RT-1^n^) implanted into Lewis rats (MHC: RT-1^l^). These rat strains are major MHC-mismatched (8). Male Lewis rats between the ages of 36-42 days from Charles River were allowed to acclimate for 72 hours after arriving at the facility then received a 1 mg/kg dose of sustained release buprenorphine SC and were anesthetized using 1-4% isoflurane in balance oxygen. Under sterile technique, a 5-10 mm incision was made on the dorsal neck region followed by a SC pouch by blunt dissection. Animals received i) cell-seeded scaffolds, ii) injections of freed cells in PBS, or iii) no implant/injection (sham). EC implanted rats received either 1x10^6^ ECs in 50 µl PBS for 2D freed or 5 scaffolds with 2x10^5^ cells/scaffold. The wound was then closed with wound clips and wound glue. No immunosuppression was given.

Acute groups were sacrificed on day 5 and chronic groups were sacrificed on day 28. In addition to the acute and chronic groups, there was also a booster group of *in vivo* implantation experiments where the rats received multiple challenges with allogeneic cells *in vivo* before isolating immune cells and running the assays *in vitro*. In the booster group, rats initially received implants of either 2Df, 3DSF, 3Df ECs or no cells (sham). After 23 days, 5 days before the end of the study, all rats including the sham group, received an injection of 1x10^6^ 2Df ECs (in 100 µl PBS, using 25-gauge needle) to stimulate a second immune response. The sham + 2Df group serves as a measure of the initial immune response to 2Df ECs.

### 2.5 Serum and splenocyte isolation

At the end of the study, rats were euthanized using CO_2_ overdose. After death was confirmed, the abdomen was sprayed with 70% EtOH, and incisions were made through the skin and muscle below the diaphragm. The diaphragm was cut and the chest cavity opened. A 21-gauge needle was inserted in to the left ventricle and between 4-8 ml blood was collected. The blood was allowed to sit undisturbed for 30 minutes, spun down at 3000 RPM for 10 min at 4°C. The serum layer was collected and frozen at -80°C until use. For animals implanted with scaffolds, the scaffold was retrieved from the SC space and fixed in 4% paraformaldehyde overnight before being transferred to 70% ethanol for histology.

The spleen was collected and placed in complete RPMI on ice. All splenocyte isolation was done in RPMI supplemented with 10% FBS, 0.1 M HEPES, and 200 U/ml penicillin-streptomycin. The spleen and 10 ml RPMI were placed in the lid of a 60 mm petri dish. The base of the dish was fitted with a cell dissociation sieve (Sigma, CD1-1KT) with a size 60 mesh (Sigma, S1020). The spleen was cut into small pieces using a size 15 scalpel. Pieces were then pushed through the mesh using a glass plunger, with frequent rinsing with RPMI from the lid. Collected splenocytes (SP) were spun down at 400 g for 5 minutes, the pellet was resuspended in 10 ml ACK buffer (Thermo, A1049201), and allowed to sit at RT for 3 minutes to lyse red blood cells. SPs were then washed and were put through a 40 µm filter. This method yielded approximately 2.5-3.5x10^8^ cells per animal. After counting, SPs were spun down, suspended in heat inactivated FBS, and frozen in 10% DMSO at 5-7x10^7^ cells/ml. Predicted composition of isolated SPs is 21-25% T cells and 44-58% B cells, with the remainder comprised of monocytes, granulocytes, dendritic cells, natural killer cells, and macrophages (9).

### 2.6 Thawing splenocytes and counting

Frozen SPs were thawed in a 37° C water bath until a small chunk of ice remained. The contents were then quickly transferred to 9 ml warm complete RPMI. The cells were spun down and resuspended in 20 ml complete RPMI. SPs were rested overnight in an incubator in 50 ml conical tubes with caps loosened. The next day, SPs were counted 1:1 in trypan blue and the live cell number was used for assay calculations. Vials frozen with 5-7x10^7^ total cells yielded 4-10x10^6^ live cells after thawing and overnight rest. SPs were then diluted to 1x10^6^ cells/ml.

### 2.7 Cytotoxicity assay

The cytotoxicity assay was run in white-walled 96-well plates. Each set of SPs (one from each rat) had 3 wells of SPs alone and 3 wells of target cell-SP co-culture. Additional controls included three wells each of target cells alone (basal cell death), target cells + 2% Triton X-100 (max cell death), and media alone. Target to effector ratios of 1:20, 1:10, and 1:5 were tested.

Diluted SPs were plated at 5x10^4^ SPs/well or 50 µl/well. Target cells (ECs) grown on TCP were lifted with trypsin which was neutralized with complete RPMI. Target cells were resuspended in serum-free media and 1x10^4^ target cells/well were added to the co-culture, target cell alone, and triton wells (100 µl/well). Additional serum-free media was added to the wells such that the final volume of each well was 200 µl. Plates were kept in the incubator undisturbed for 3 hours. The ratio of target to effector cells was 1:2. Reagents from the CytoTox-Glo Cytotoxicity Assay (Promega, G9291) were prepared as described in the manual. After 3 hours, 50 µl of the CytoTox-Glo Cytotoxicity Assay Reagent was added to each well. Wells were mixed briefly by orbital shaking and then incubated for 15 minutes at room temperature. Luminescence was measured for 1 second/well.

Luminescence from cell death was determined by subtracting the luminescence for the SP alone wells from the SP-target cell co-culture wells to correct for spontaneous cell death. Values greater than zero indicate greater cell death in the co-cultured wells while values less than zero indicate greater cell death in SP alone wells.

### 2.8 Proliferation assay and FlowJo analysis

The proliferation assay was run in 96-well, round bottom plates. Each set of SPs (one from each rat) was run in triplicate with 3 wells of SPs alone and 3 wells of target cell-SP co-culture. Additional controls included unstained SPs and single-color controls. Target to effector ratios of 1:10, 1:5, 1:2 were tested. Comparisons of freshly isolated and thawed frozen SPs were tested to ensure freezing did not influence proliferation.

For 2-day co-culture proliferation experiments, target cells were stimulated with 40 ng/ml rat recombinant IFNγ (R&D Systems, 585-IF-100) for 48 hours before use in the assay. Target cells in media without added rrIFNγ were used for 4-day co-culture proliferation assays. Target cells were lifted using trypsin, treated with 50 µg/ml mitomycin C (Sigma, 1010749001) for 30 minutes to prevent proliferation, and rinsed twice before plating.

Diluted SPs were plated at 1x10^5^ SPs/well or 100 µl/well. A 1:2000 CellTrace Violet Dye (Thermo, C34557) was made up in PBS + 0.1% heat inactivated fetal bovine serum (FBS). SPs were spun down at 400 g for 5 min and stained with CellTrace Dye per manufacturer instructions. Target cells were diluted to 5x10^5^ cells/ml and 100 µl (5x10^4^ cells) was added to each well. The ratio of target to effector cells was 1:2. Additional complete RPMI was added to the SP alone and control wells such that each well had a final volume of 200 µl. Plates were incubated in the dark for 2 or 4 days without disturbing.

After incubation, plates were spun down at 400 g for 5 minutes and resuspended in flow buffer (PBS + 2% heat inactivated FBS) with a 1:400 dilution of FITC Mouse Anti-Rat CD3 (BD, 557354). Plates were incubated on ice for 1 hour, protected from light before washing and resuspension in flow buffer. Single color compensation controls for FITC channel were created using UltraComp eBeads (Thermo, 01-2222-41) and well as single color controls of SPs with only CellTrace Violet or only CD3-FITC. Plates were run on a BD FACSCanto II Cell Analyzer. A least 1x10^4^ size gated, CD3+ cells were collected and analyzed from each well. As the cells proliferate, CellTrace Violet fluorescence decreases. Proliferation was determined by subtracting the mean fluorescence in the CellTrace channel of SP-target cell co-culture wells from SP alone wells, for CD3+ cells only.

### 2.9 IFNγ ELISA

Supernatant from the proliferation assay plates was collected on day 2 or day 4 and frozen at -20°C until ready for analysis. The supernatant was run at full concentration (100 µl/well) with a standard run in duplicate using an IFN gamma Rat ELISA kit (Thermo, BMS621). The standard curve was fit using a 4-parameter logistic curve in Prism Graphpad. IFNγ at 37°C has a half-life of approximately 24 hours and is stable when frozen at -20°C for several months.

### 2.10 Serum assay

Serum samples stored at -80° C were allowed to come to room temperature. Serum was decomplemented by incubating at 56°C for 30 minutes. The serum assay was run in 96-well, round bottom plates. ECs were plated at 1x10^5^ cells in 50 µl per well along with 50 µl of decomplemented serum (1:1 ratio). Previously, serum ratio of 1:2, 1:5, and 1:10 were tested before it was determined that 1:2 showed the best signal. Each sample of serum was run in duplicate with controls of cells with serum only, with antibody only, and with neither serum nor antibody. After the addition of serum, plates were incubated on ice for 1 hour, washed twice and labeled with 0.125 µg/ml (1:160 dilution) BV421 Mouse Anti-Rat IgM (BD, 742499), and 0.25 µg/ml (1:80 dilution) APC Mouse anti-rat IgG1 (Thermo, 17-4812-82) for 1 hour on ice, protected from light. Single color compensation controls for FITC channel were created using UltraComp eBeads (Thermo, 01-2222-41). Plates were run on a BD FACSCanto II Cell Analyzer. A least 1x104 CD3+ cells were collected and analyzed from each well.

### 2.11 Histology

*In vitro* cultured scaffolds and explanted scaffolds were fixed with 4% paraformaldehyde overnight and then submitted in 70% EtOH to the Koch Histology Core for paraffin embedding, sectioning, staining, and slide scanning. H&E stains and immunohistochemical (IHC) stains were carried out by the Histology Core. Slides were sectioned at 5 µm. IHC was run on the ThermoScientific IHC Autostainer 360 with 10 min for endogenous peroxidase blocking, 30 minutes for protein block, 60 minutes for primary antibody, 15 minutes for labeled polymer, and DAB for 10 minutes. IHC stained were used at 1:50 for the rabbit ant-CD31 (Thermo, PA5-32321) and 1:200 for the rabbit anti-HIF1a (abcam, ab216842).

### 2.12 Statistical analysis

Statistical analysis was carried out using Graphpad Prism using ordinary one-way ANOVA and 2-way ANOVA for multiple comparisons as noted in the figure captions.

## 3 Results

### 3.1 Substrate interactions modulate endothelial cell response to cytokines

**Figure 1:**
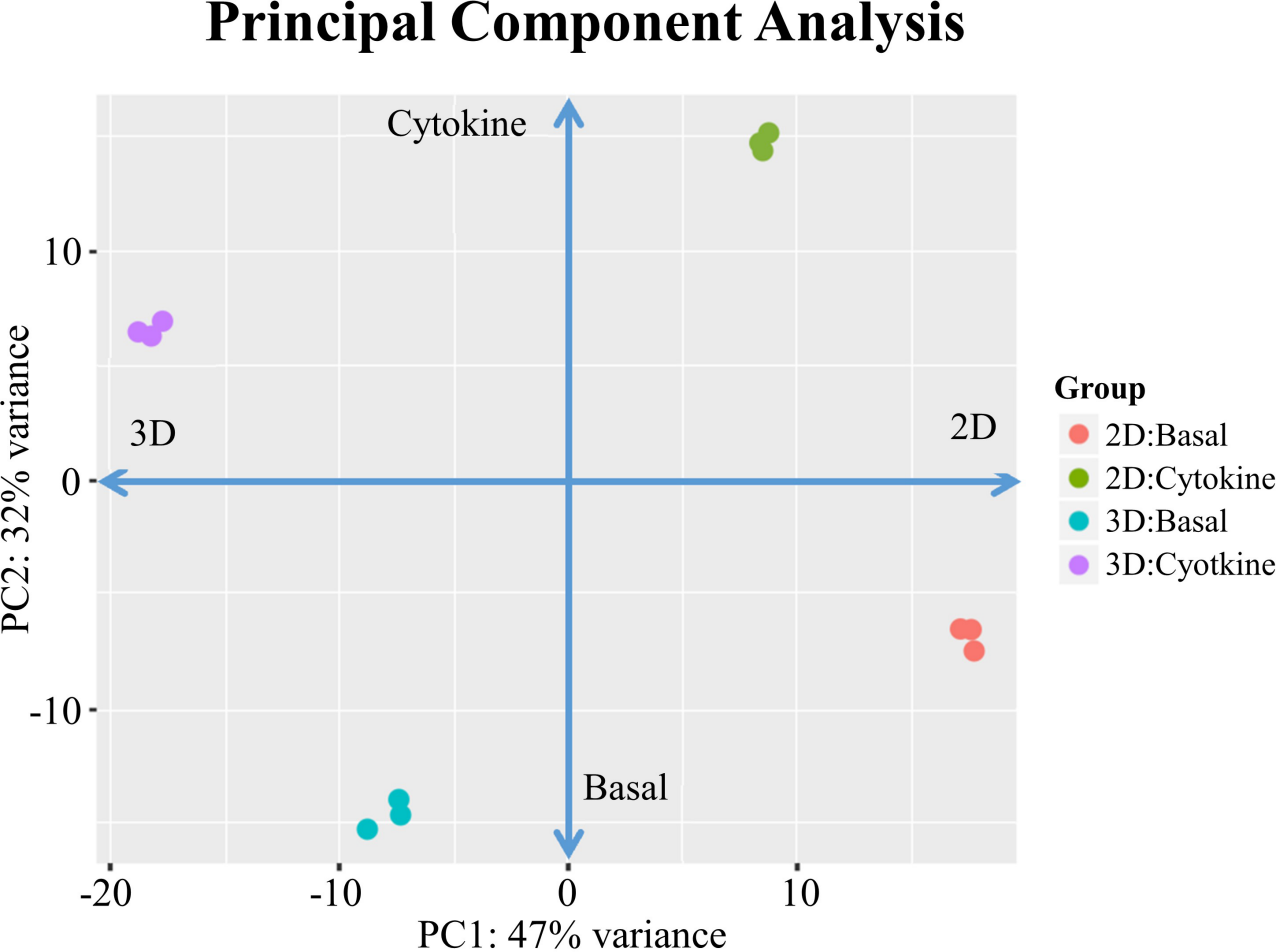
PCA for 2D confluent and d3/d14 3DSF ECs. PCA shows that treatment with cytokines significantly shifts gene expression in both 2D and 3D ECs, although each maintain their unique substratum-associated phenotypes.

We evaluated whether EC-substratum interactions influenced the ability of ECs to respond to external stimuli, focusing on specifically on response to cytokines. ECs were cultured with IFNγ and TNFα, two cytokines which synergistically increase EC activation (10). A PCA plot of basal and cytokine-treated 2D and 3D ECs demonstrates that the addition of cytokines dramatically changes the gene expression of cultured ECs cultured, shifting their patterns of gene expression while still maintaining the observed differences between 2D and 3D culture conditions (**Figure 1**). Cytokines did not drive 2D and 3D ECs towards a common “cytokine activated” state. While the expression of several genes was similar in the basal condition, 3D ECs were generally more responsive to the addition of cytokines than 2D ECs (**Supplementary Figure 1**).

Cytokine treated 3D ECs demonstrated upregulation of cell adhesion and vascular effects while cytokine treated 2D ECs upregulated of surface receptors (**Supplementary Figure 2**). In general, the addition of cytokines to 2D ECs increased the gene expression of intracellular signaling, inflammatory cytokines, and pattern recognition receptors (PRRs) while decreasing the expression of ECM remodeling associated genes. Interestingly, 2D cytokine-treated ECs upregulated genes associated with cell cycle arrest and apoptosis (**Supplementary Figure 3**).

We confirmed that the addition of cytokines did not alter our previous study observations of a hypoxic 3D EC phenotype and a 2D endothelial to mesenchymal transition (EndMT) phenotype (**Supplementary Figure 4A, B**). With the use of GO gene sets related to IFNγ and TNFα, we confirmed that matrix-embedding does not shield the cells from circulating factors. (**Supplementary Figure 4C**).

### 3.2 Cytokine induced differences in immune cell recruitment, leukocyte adhesion, antigen presentation, and co-stimulation

While there are substratum-determined differences in basal, quiescent ECs, the full dynamic range of the differences in 2D and 3D is more evident when ECs are exposed to the inflammatory cytokines IFNγ and TNFα. Several functions of ECs, such as promoting immune cell transmigration, MHC class II presentation, and pro-inflammatory paracrine signaling are only observable in activated ECs (11, 12). We chose to focus on four specific mechanisms by which ECs function to modulate immune responses (**Supplementary Figure 5**). We broadly looked at genes associated with (a) leukocyte recruitment through the release of chemokines, (b) leukocyte rolling and adhesion, (c) antigen processing and presentation, and (d) co-stimulation and inhibition.

From these categories, we identified three trends (**Table 1**): i) genes which were expressed in the basal state and upregulated by both 3D culture and cytokine treatment, ii) genes which were not expressed in the basal state and when induced in the cytokine treated state, were more significantly upregulated in 3D ECs, and iii) co-stimulatory and inhibitory molecules where upregulation and downregulation was mixed and depended on the specific EC state.

**Table 1:** Summary of gene expression changes in adhesion, MHC, and co-stimulatory molecules.

	Gene	Change due to cytokines		Change due to 3D culture	
		2D	3D	Basal	Cytokine
(A)	ICAM1	↑	↑	↑	↑
	RT1-A1	↑	↑	↑	↑
	RT1-A2	↑	↑	↑	↑
	PD-L1	↑	↑	↑	↑
(B)	VCAM1	–	–	–	↑
	RT1-Ba	–	–	–	↑
	RT1-Bb	–	–	–	↑
	HVEM	–	–	–	↑
(C)	CD137	–	↓	↑	–
	CD48	↓	–	–	–
	ICOSL	↓	–	↑	↑
	CD80	↑	↑	–	↓

RNAseq of ECs showed three patterns of response to 3D culture and cytokine stimulation: (A) genes expressed in the basal state and upregulated by both 3D culture and cytokine treatment, (B) genes not expressed in the basal state and only upregulated in cytokine treated 3D ECs, and (C) co-stimulatory and inhibitory molecules where upregulation and downregulation was mixed and depended on the specific EC state.

#### 3.2.1 Constitutive expression and consistent upregulation by 3D culture and cytokine treatment

**Figure 2:**
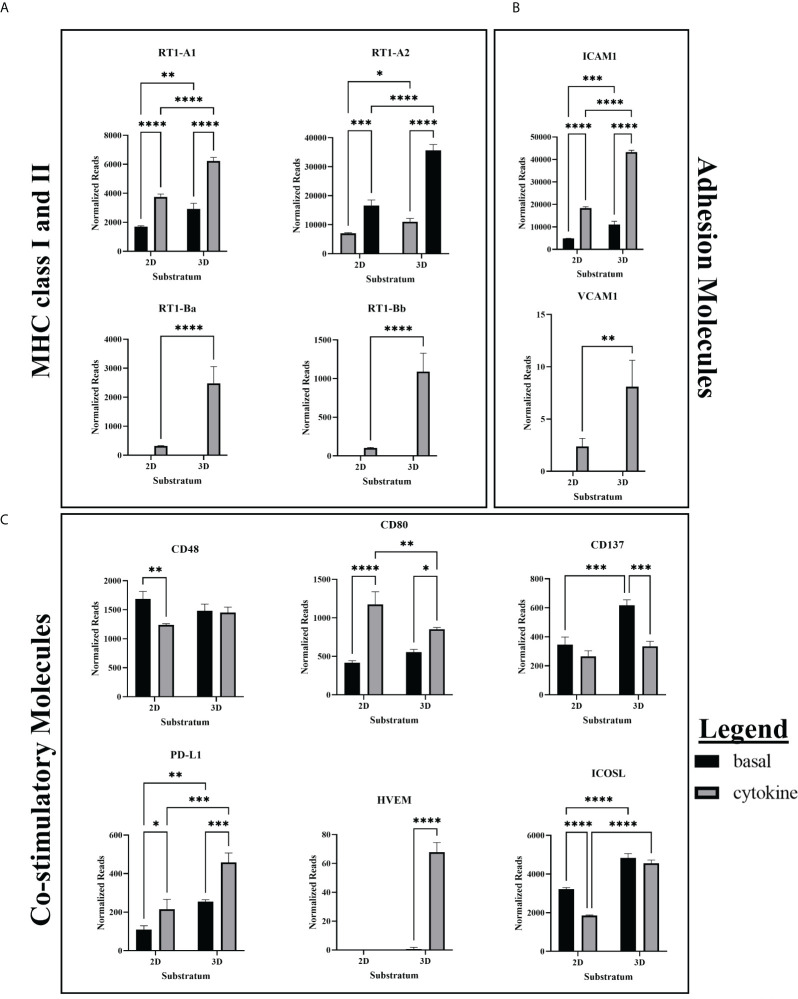
RNA expression of MHC, co-stimulatory, and adhesion molecules. **(A)** RNAseq showed greater basal expression and upregulation of MHC class I and class II in response to cytokine stimulation in 3D ECs. **(B)** RNA Expression of ICAM1 and VCAM1 is upregulated in cytokine-treated and 3D cultured ECs. **(C)** CD80 and PD-L1 were constitutively expressed while HVEM was induced by cytokine treatment. Cytokine treatment decreased expression of CD137 in 3D ECs, and CD48 and ICOSL in 2D ECs. RNAseq cytokine ECs were treated with IFNγ and TNFα. Statistics: RNA – two-way ANOVA. n = 3. *(P ≤ 0.05), **(P ≤ 0.01), ***(P ≤ 0.001), ****(P ≤ 0.0001).

ICAM1, RT1-A (MHC class I), and PD-L1 were all constitutively expressed in the basal state and increased their expression with cytokine treatment (**Figure 2**). Further, all showed statistically significant upregulation when cultured in 3D as opposed to 2D. These three molecules are involved in essential EC functions which help maintain vascular and immune homeostasis. Leukocyte adhesion through ICAM-1 plays an important role in the formation of immune synapses between APCs and T cells (13). Additionally, ICAM-1 is essential to stable arrest of rolling immune cells on high endothelial venules in the lymph node, which is essential for extravasation into the tissue (14).

Basal state ECs constitutively express MHC class I and the TAP proteins required for MHC class I peptide transport, and both are upregulated with IFNγ stimulation (3). However, we found that MHC class I and the associated TAP proteins are more significantly upregulated by cytokine stimulation in 3D ECs when compared to 2D ECs (**Supplementary Figure 6**). Differences in MHC class I and TAP expression between 2D and 3D ECs may affect their growth rate and responsiveness to paracrine signaling (15).

PD-L1 functions as an inhibitory molecule which induces immune exhaustion, effector T cell apoptosis, inhibits T cell replication and maturation, and induces of development of T regulatory cells (Tregs) (16). It is constitutively expressed in resting lymphocytes and APCs and induced during inflammation in endothelial and epithelial cells (16). More specifically, PD-L1 is upregulated in IFNγ and TNFα stimulated ECs, preventing CD8+ T cell activation and cytotoxic function (17). As previously described in the literature, our model demonstrated that both 2D and 3D cytokine treated ECs upregulated their expression of PD-L1 (**Figure 2**).

#### 3.2.2 Genes upregulated only in cytokine treated 3D ECs

VCAM1, RT-1B (MHC class II), and HVEM were only expressed in cytokine treated ECs and were significantly upregulated in 3D ECs when compared to 2D ECs (**Figure 2**). These three molecules are related to blood vessel EC-specific functions in inflammation or infection. When activated, ECs have the unique ability among parenchymal cells to express MHC class II in addition to MHC class I (3). VCAM1 and ICAM1 are essential for adhesion of immune cells to the vascular endothelium and leukocyte-EC signal transduction (3). While ICAM-1 is constitutively expressed, VCAM-1 is preferentially expressed during Th2 CD4+ induced inflammation (18, 19).

HVEM interacts with three different receptors (BTLA, HVEM, LIGHT) which prevents excessive tissue inflammation (**Figure 2**). Generally, BTLA and CD160 function as an inhibitory receptors, modulating T cell activation (20). T cells can transiently express membrane-bound LIGHT which acts as a co-stimulatory receptor when it interacts with HVEM (20). In our culture system, only the cytokine treated 3D ECs expressed significant amounts of HVEM, providing another possible mechanism for EC immunomodulation. However, CD160 is also expressed in newly formed blood vessels so upregulation of HVEM could also be associated with signaling between neighboring ECs during angiogenesis.

#### 3.2.3 Genes differentially expressed across conditions

CD48, ICOSL, and CD80 showed mixed patterns of upregulation and downregulation depending on specific group comparisons (**Table 1**). These signals vary both spatially and temporally. Determining the effect of these proteins is further complicated by the fact that effector and regulatory T cells share many of the same co-stimulatory and co-inhibitory molecules, which are expressed differently after immune activation (21). For example, CD4+ T cells increase their expression of ICOS and OX40 when co-cultured with ECs. Blocking these co-stimulatory molecules significantly decreases T cell proliferation (22).

CD80 is the ligand for both CD28 and CTLA-4 on T cells. Although CD28 and CTLA-4 bind the same receptors and are expressed on the same cells, the general convention is that CD28 is co-stimulatory while CTLA-4 is an inhibits T cell function. It has been proposed that CD80-CTLA4 interactions important for maintaining self-tolerance although, the functional outcome appears to be largely dependent on timing and context (23). In our culture system, CD80 expression patterns were mixed (**Figure 2**). Differences in responses to cytokines, lack of expression in human ECs, and an incomplete understanding of the downstream outcomes of CD80 signaling make it difficult predict the effect of their expression will have on immune recognition in ECs.

Surprisingly, our studies showed that that CD48 expression was not increased by IFNγ stimulation as the literature would suggest, but decreased as a result of cytokine stimulation in 2D ECs (**Figure 2**). As CD48 is only expressed in immune cells and ECs, the addition of cytokines could further promote EndMT in 2D ECs, moving them away from the EC phenotype and towards non-CD48 expressing mesenchymal cells.

In ECs, ICOSL serves as a co-stimulatory molecule (24, 25). Our results show that in the 2D cytokine condition, ICOSL expression was reduced, likely due to increased EndMT and loss of the EC phenotype that allows for ICOSL expression, as was observed in CD48 (**Figure 2**). While cytokine stimulation did not increase ICOSL expression in 3D ECs, in both the basal and cytokine conditions, 3D ECs had significantly greater expression of ICOSL than 2D ECs.

#### 3.2.4 Chemokines

ECs can express a range of different chemokines although they release minimal amounts in their basal, unstimulated state (26). In response to inflammation (hypoxia, infection, or oxLDL), ECs upregulate their production of chemokines (27). The release of chemokines by ECs allows them to orchestrate the movement of immune cells into the tissue to combat infection and promote tissue repair. In the cytokine-treated condition, both 2D and 3D ECs upregulated their chemokine expression but interestingly, differed in which chemokines were upregulated (**Supplementary Figure 7**).

In summary, cytokine stimulation and 3D culture uniformly increase gene expression (**Table 1**). This trend is also seen in HVEM and PD-L1 which generally inhibit T cell activation, and CD86 which can be inhibitory when interacting with CTLA-4. The predominantly co-stimulatory proteins CD48 and ICOSL show a trend of upregulation with 3D culture and downregulation in response to cytokine stimulus. Lastly, CD80 which acts as a co-stimulatory molecule through its interaction with CD28, shows upregulation when ECs are exposed to cytokines, although the increase is smaller in 3D ECs.

### 3.3 Development of *in vivo* transplantation model

**Figure 3:**
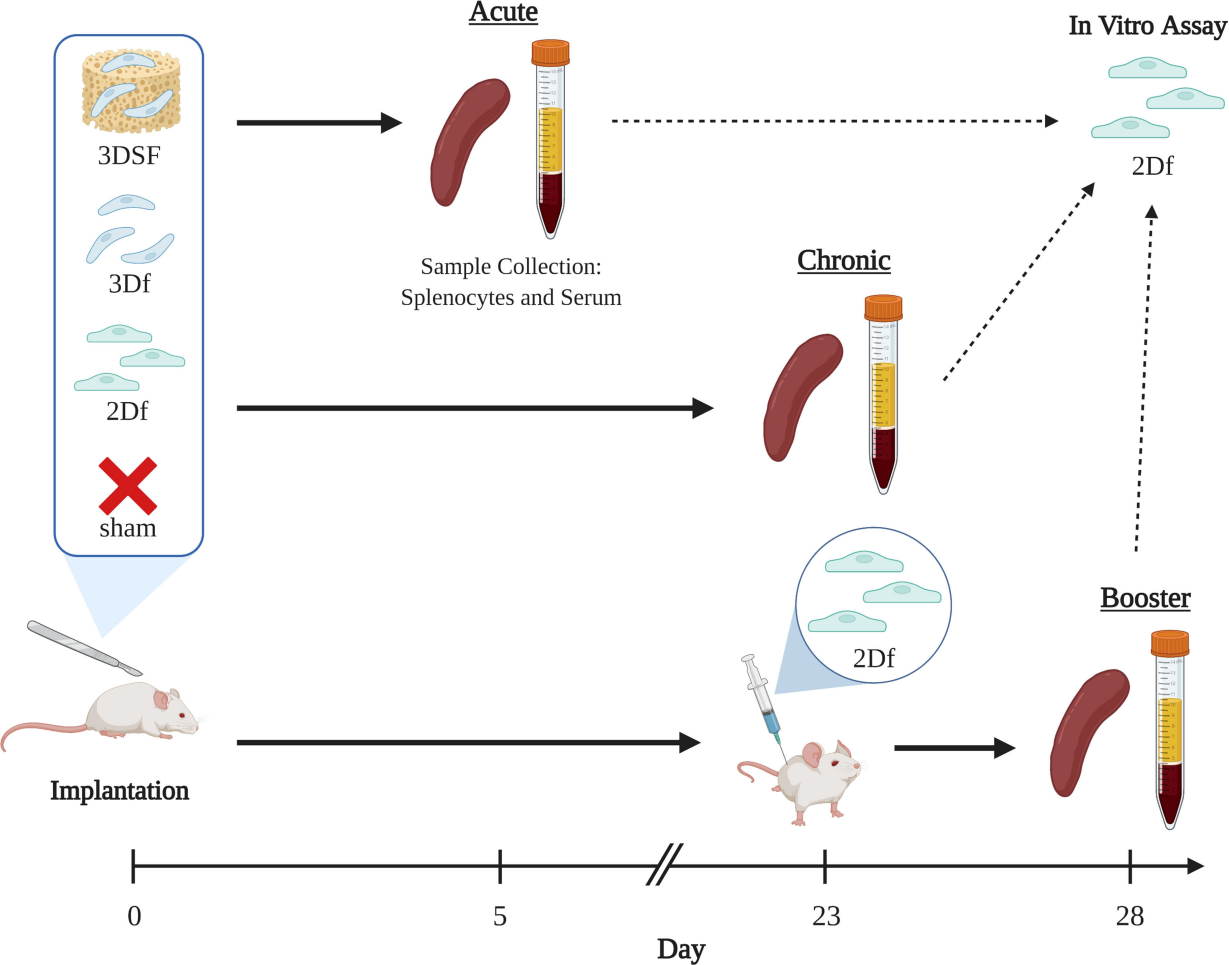
Experimental groups and time scales for *in vivo* studies. The effect of implantation of allogeneic ECs on immune response was measured over three different time scales. Brown Norway ECs were implanted subcutaneously in Lewis rats. Animals were implanted with either i) 2D ECs freed from TCP with trypsin (2Df), ii) 3D ECs that were implanted on their scaffolds (3DSF), iii) 3D ECs freed from the scaffold with trypsin (3Df), and iv) surgery but no implants (sham). Acute animals were implanted for 5 days and chronic animals were implanted for 28 days. Booster animals received either implants or sham surgery at day 0 and were all given injections of 2D freed ECs at day 23. Created with BioRender.com.

To understand the role of substratum interactions in transplantation, we studied the immune response to subcutaneous implants of allogeneic ECs over several different *in vivo* time scales (**Figure 3**). First, we used an *in vitro* co-culture model to confirm that measured responses were allogeneic and due to strain-mismatch (**Supplementary Figure 8**). We isolated immune cells during two stages of the immune response: i) the acute phase (5 days after implant), which the effector phase of clonal expansion and differentiation, and ii) the chronic phase (28 days after implant), after immune cell contraction. In both cases, splenocytes (SPs), primarily T and B cells, isolated from the implanted animals were re-exposed to the original EC stimulus *ex vivo*. In the booster group, similar to the chronic group, animals were sacrificed after 28 days, however, 5 days before the end of the study, all animals received additional 2D EC injection to elicit a secondary response *in vivo*. This additional challenge shows to the extent to which the rats’ immune systems had retained a memory response to the allogeneic ECs after returning to homeostasis.

Transplanted cells are generally implanted either as cells in suspension (for example, stem cell therapies) or cells adhered to a matrix (organ or tissue transplants). The 3D cultured ECs (referred to as 3D Surgifoam or 3DSF) had the advantage of being easily storable, transportable, and implantable, retaining the matrix adherence and phenotypic features developed in culture. However, it was not possible to similarly implant 2D TCP ECs while still adhered to their substratum. Instead, 2D ECs were lifted from the TCP (2D freed or 2Df) using trypsin and implanted in a suspension. To control for differences in immune response to cells in suspension compared to matrix-adherent cells, we also lifted 3D ECs from their scaffolds (3D freed or 3Df) and implanted them in suspension. The 3D freed group differentiates between the physical effects of surface adherence and the changes in EC phenotype that occur during culture. Finally, the naïve control group had a sham surgery without the implantation of cell or scaffolds.

Using a combination of mixed lymphocyte assays and supernatant ELISAs, we characterized the immune response to allogeneic and syngeneic ECs both *in vitro* and *in vivo* over multiple time scales.

### 3.4 Cytotoxic response to allogeneic cellular implants

**Figure 4:**
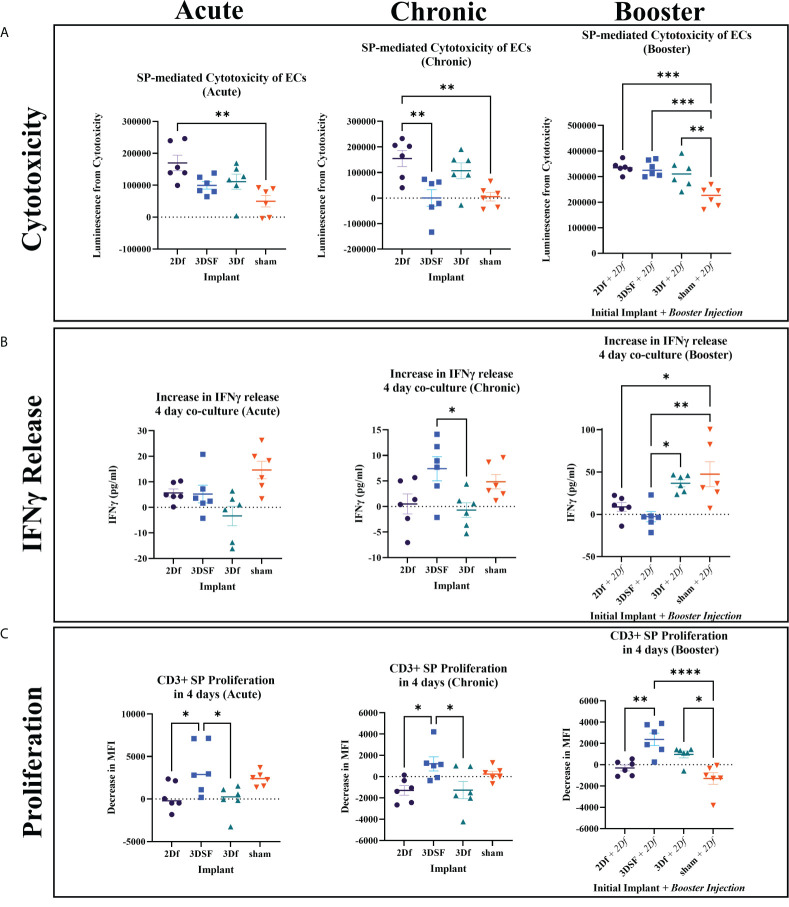
*In vivo* assays measuring cytotoxicity, IFNγ release, and proliferation from SPs isolated from rats implanted with allogeneic ECs. **(A)** Cytotoxicity was elevated for 2Df EC implanted rats relative sham for all groups while 3DSF 3D implanted acute and chronic rats had lower cytotoxicity. **(B)** 3DSF and sham SPs showed elevated IFNγ release in co-culture wells compared to SP alone wells while 2Df and 3Df did not. At day 4, SPs from 3Df and sham animals showed significantly elevated IFNγ release in co-culture wells after a second *in vivo* injection of 2Df ECs. **(C)** MFI, mean fluorescence intensity. 3DSF SPs showed greater proliferation than 2Df and 3Df SPs in both the acute and chronic groups. 3DSF and 3Df SPs show greater proliferation than sham SPs when rats receive a booster injection of 2Df ECs. Statistics: Ordinary one-way ANOVA. n=6. *(P ≤ 0.05), **(P ≤ 0.01), ***(P ≤ 0.001), ****(P ≤ 0.0001).

SPs isolated from sham rats showed minimal cytotoxic effects (**Figure 4A**). In cases such as the chronic group, where ECs remained implanted for longer periods of time, co-culture of allogeneic ECs with sham SPs resulted in negligible EC killing. EC death was slightly elevated in co-culture with acute sham SPs, likely due to a general inflammatory response from the sham surgery.

In both the acute and chronic setting, implantation with 2Df ECs resulted in a statistically significant increase in cytotoxic ability of isolated SPs. In the acute setting, neither the 3DSF or 3Df groups showed a statistically significant increase over sham, indicating that ECs with a 3D phenotype caused less of a cytotoxic response than 2D ECs. In the chronic setting, SPs isolated from rats that received an implant of 2Df cells still caused the greatest amount of cytotoxicity, being statistically greater than 3DSF implanted and sham animals. In summary, implantation with freed ECs caused similar cytotoxic effects in both acute and chronic setting. Rats exposed to 2D ECs were able to rapidly mount a cytotoxic response *ex vivo* even 28 days post implantation. For implants of 3DSF ECs, cytotoxic ability decreases over time post implantation, even though 3DSF ECs are presumably retained in the animals for longer since they are adherent to a scaffold. The memory response in animals implanted with 3DSF ECs is not as robust as in animals implanted with 2D ECs.

In the booster animals, the sham + 2Df group had a cytotoxic response similar to the 2Df group in the acute setting. The animals who originally received an implant of 2Df, 3DSF, and 3Df ECs had an even greater immune response both in terms of their absolute value (luminescence) and relative to animals which only had one implant of ECs. Comparing this to the results from the acute and chronic animals, we found that while the cytotoxic effect from 3D ECs decreases over time, repeat challenge with 2Df ECs elicits a strong memory response.

### 3.5 *In vitro* release of IFNγ in co-cultured splenocytes

IFNγ release from SPs isolated from acutely implanted rats was measured after 2 and 4 days in co-culture. After 2 days of co-culture, all three groups that received implants of ECs showed greater IFNγ release in the co-cultured wells while the sham group did not (**Supplementary Figure 9**). The elevated response of the animals that were implanted with ECs relative to sham rats is similar to what was seen in the acute cytotoxicity assay. After 4 days in culture, co-cultured SPs from rats implanted with ECs had decreased their release of IFNγ to a similar level as SPs that were not stimulated. On the other hand, co-cultured sham SPs increased their release of IFNγ. *In vitro* co-culture with allogeneic ECs can stimulate allogeneic SPs to produce IFNγ, even in the sham case where the rats were not previously exposed to allogeneic ECs.

IFNγ is reported to have a half-life *in vivo* ranging from 30 min to 4.5 hours, thus IFNγ concentration measured at day 4 is not expected to be cumulative but rather a measure of recent IFNγ release (28). As a result, highly cytotoxic SPs from rats implanted with ECs that were effective at killing ECs *ex vivo* likely removed or reduced the allogeneic stimulus and resulted in a decreased measurement of IFNγ by day 4. Sham rat SPs likely took longer to respond to the ECs since this was their first exposure and started increasing their IFNγ production after several days in culture. IFNγ release for chronic SPs was measured after 4 days in co-culture and shows a similar inverse relationship with their cytotoxicity results (**Figure 4B**). By day 4, SPs from the two freed groups did not release more IFNγ in the co-culture wells compared to their respective SP alone wells. In contrast, the 3DSF group, which did not have a large cytotoxic response, was releasing IFNγ 4 days into the EC-SP co-culture, significantly more than the 3D freed group.

Finally, IFNγ in the booster assay was also measured after 2 and 4 days of co-culture. The 2 day co-culture IFNγ results for the booster group were similar to the results for acutely implanted rats, showing that all booster animals responded to the additional injection of 2Df ECs (**Supplementary Figure 9**). As in all the other 4 day co-culture groups, the sham group was elevated. Release of IFNγ from SPs isolated from 3Df implanted rats remained high after 4 days in culture.

In summary, after 4 days of co-culture, the 2Df group consistently exhibited low IFNγ release in the co-cultured wells while the sham group had consistently elevated IFNγ release in the co-cultured wells. The greatest differences between the IFNγ release over the three different time scales of *in vivo* experiments were observed between the 3DSF and 3Df ECs highlighting the potential role of the physical barrier of the scaffold as opposed to solely substratum related gene expression changes.

### 3.6 Proliferative response to allogeneic cellular implants

T cell proliferation, gated by size and CD3+ expression, was measured at 2 days and 4 days of co-culture (**Figure 4C**, **Supplementary Figure 10**). Despite differences in cytotoxicity and IFNγ release between acute and chronic time scales, the proliferative response of SPs from these two different time scales showed similar relationships. After 4 days of co-culture, the 3DSF SPs from both experiments had a greater proliferative response than the 2Df or 3Df SPs. In general, the co-cultured SPs from the freed EC groups did not proliferate more than unstimulated SPs. Meanwhile, sham SPs proliferated when co-cultured with allogeneic ECs, as was seen in the naïve allogeneic control experiments (**Supplementary Figures 10**, **11**). The proliferation of 3DSF SPs is generally similar to proliferation in sham SPs. The differences in the immune response between the cytotoxic assay and the proliferation assay demonstrate that general T cell proliferation is not always directly equivalent T cell cytotoxic ability.

Proliferation results from the booster group show that while 3DSF SP proliferation was similar to sham proliferation in the animals that received only one allogeneic stimulus, previous implantation with 3DSF ECs changed the way the immune system responded to a subsequent injection of 2Df ECs (**Figure 4C**). In the booster proliferation assay, 3DSF and 3Df SP proliferation was elevated over the sham group. While 3DSF SP proliferation was elevated relative to 2Df SPs, it was not statistically greater than 3Df ECs like it was in the acute and chronic assays.

In summary, SPs from 3DSF and sham animals were able to proliferate *in vitro* when challenged with allogeneic ECs whereas SPs from rats initially implanted with 2Df and 3Df SPs did so only after a second *in vivo* injection. Also, a second *in vivo* challenge with allogeneic ECs creates a greater level of proliferation than a single *in vivo* challenge with 2Df ECs.

### 3.7 Serum response to chronic implants

**Figure 5:**
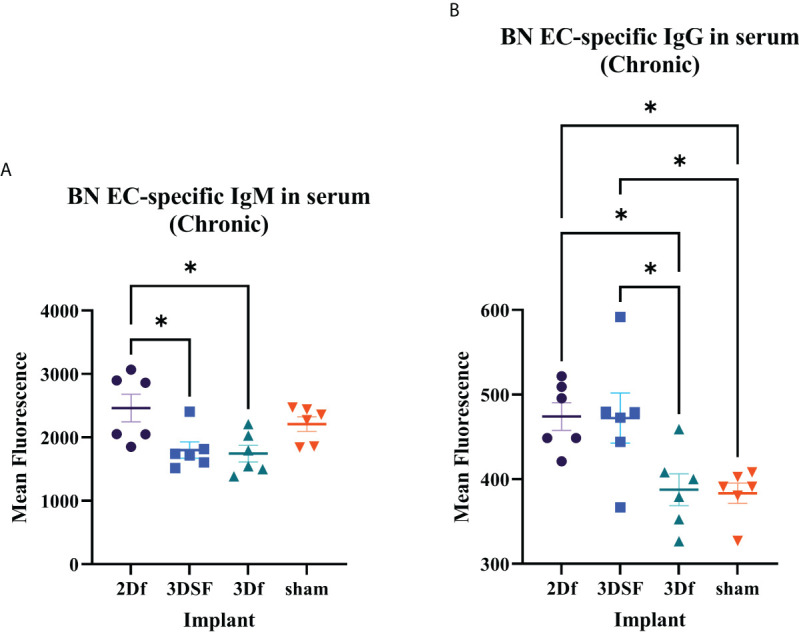
Serum antibody levels in rats chronically implanted with allogeneic ECs. Measurement of BN EC-specific antibodies. **(A)** There was greater circulating IgM in 2Df and 3DSF EC-treated rats. **(B)** Serum from 2Df and 3DSF EC-treated rats had more IgG than rats implanted with 3Df ECs or no cells. Statistics: Ordinary one-way ANOVA. *(P ≤ 0.05).

In addition to measuring T cell cytotoxicity, proliferation, and IFNγ release, we also measured the amount of BN EC-specific antibody in the serum of chronically implanted rats (**Figure 5**). We focused on IgM and IgG in serum from chronically implanted animals since it could take longer than 5 days for an appreciable level of implant-specific antibody to be detected depending on the sensitivity of the assay. Generally, IgM peaks earlier than IgG and is maintained at a much lower level over time than IgG.

In both IgM and IgG, serum antibody levels were elevated in 2Df implanted rats and low in 3Df and sham rats. Thus, previous culture condition drives differences in serum antibody levels in freed ECs. In contrast, while IgM was low in 3DSF rats, serum anti-BN IgG levels were elevated. In this case, matrix-adherence results in a differential IgM and IgG response. Comparing the relationship between the 3DSF and 3Df groups, we see that in both acute IFNγ release and IgM assays, there was no statistically significant differences between the 3DSF and 3Df groups whereas in both the chronic IFNγ release and IgG assays, the 3DSF group was significantly greater than the 3Df group.

### 3.8 Histological analysis of implanted scaffolds

**Figure 6:**
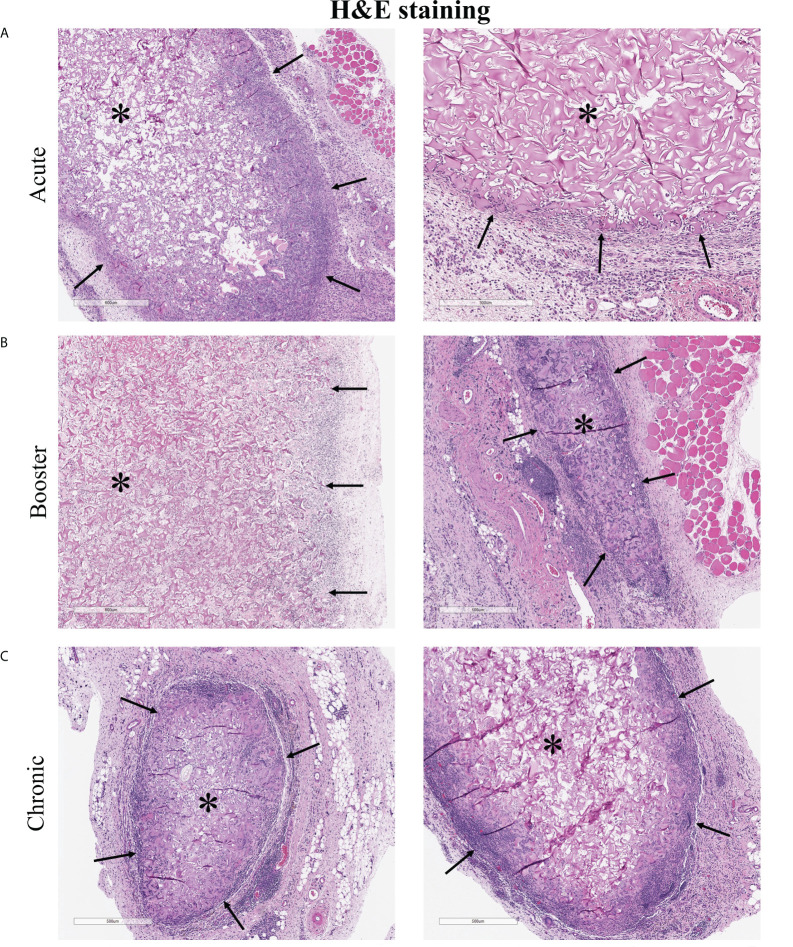
H&E staining of explanted 3D cellularized scaffolds. **(A–C)** Histology shows immune cell infiltration, fibrous encapsulation, and increased vascularization in and around scaffolds. Immune cells penetrated 165 ± 66 µm into the acute scaffolds and 227 ± 28 µm into the chronic scaffolds. Stars (*) denotes encapsulated scaffolds surrounded by immune cells, black arrows (→) point to scaffold-tissue interface and the direction of infiltrating host cells. Images from two different rats, representative of six rats per time scale.

At the end of each study, the subcutaneously implanted 3D scaffolds were excised for histological analysis to gain a gross view of the cellular reaction to the implants (**Figure 6**). Scaffolds were encapsulated and adherent to the inner surface of the skin. Acute scaffolds were much larger in size than booster and chronic scaffolds, likely due to degradation of the matrix over time, contraction with ingrowth of cells, and restriction by the fibrous capsule. Immune cell infiltration was densest at the periphery and became sparser toward the center of the scaffold. In the acute scaffolds, infiltrating cells penetrated 165 ± 66 µm into the scaffold from the edge of the fibrous capsule (20 measurements, 2 separate samples). After 23 additional days, immune cells had advanced a total of 227 ± 28 µm into the scaffold but infiltration was restricted. Even at the longest time points examined (the chronic implants) the center of the scaffolds remained largely devoid of immune cells.

**Figure 7:**
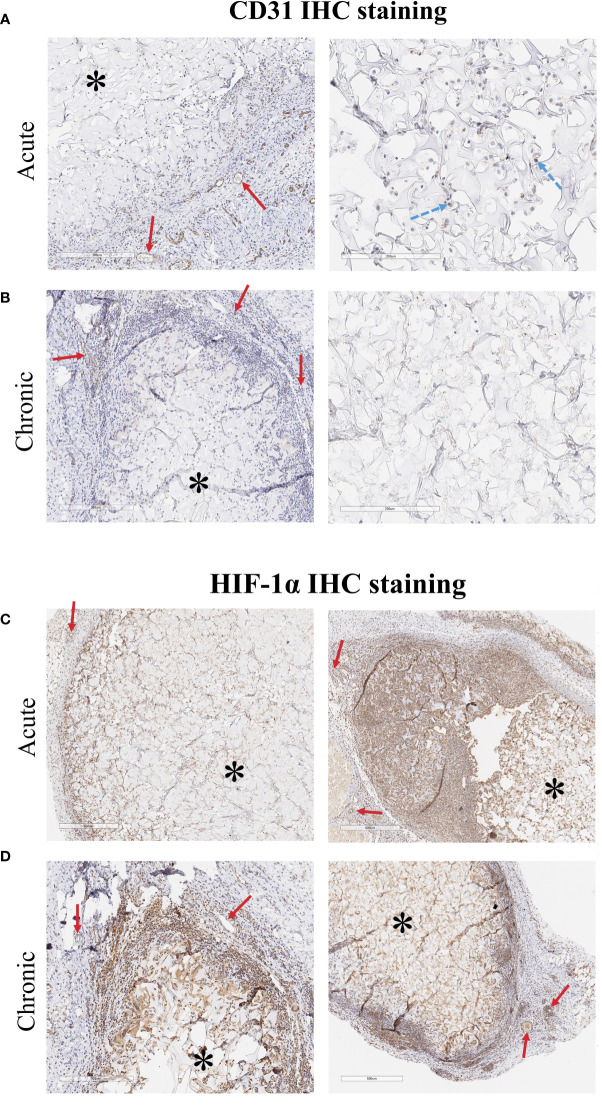
CD31 IHC staining of explanted 3D cellularized scaffolds. **(A)** Staining (brown color is CD31+ cells, hematoxylin counterstain) shows that CD31+ ECs were retained in the scaffold for at least 5 days after implant. **(B)** After 28 days, CD31+ ECs are not detectable in the areas of the scaffold that have not been infiltrated by immune cells. Staining shows increased vascularization around both the acute and chronic scaffolds. HIF-1α IHC staining of explanted 3D cellularized scaffolds. **(C, D)** HIF-1α staining (brown color is HIF-1α+ cells, hematoxylin counterstain) shows that implantation of 3D matrices creates a hypoxic region within the tissue. Immune cells that infiltrated into the scaffold also stain positive for HIF-1α. Vessels stained for HIF-1α due to the pathway’s role in angiogenesis. Images from two different rats, representative of six rats per time scale. Stars (*) denote encapsulated scaffolds surrounded by immune cells, solid red arrows (→) point to vessels along the outer edge of the implanted scaffold, dashed blue arrows (→) point to ECs embedding within the scaffold.

CD31 IHC staining helped determine whether implanted ECs survived in the implants (**Figure 7A, B**). CD31+ cells were retained in the acute implants (5 days *in vivo*) but rare if detectable at all in the chronic implants (28 days *in vivo*). CD31 staining also showed increased vascularization at the periphery of the implanted scaffolds. IHC staining of HIF-1α, a marker of hypoxia, showed that cells in and around the scaffold were hypoxic (**Figure 7C, D**). Despite increased angiogenesis into the scaffold, the hypoxic environment likely prevented immune cells from penetrating further into the scaffold and reduced the long-term viability of 3D scaffold ECs.

## 4 Discussion

Substratum is an essential component of the EC microenvironment, allowing them to retain their position at the luminal interface and serve as screening boundary cells. Substratum integrity and state also helps drive EC biology, for example, driving responses to stressors such as cytokines. We found that ECs grown on different substratums have markedly different responses to cytokine treatment. ECs retain the major substratum-induced differences observed in basal culture, including endothelial to mesenchymal transition in 2D ECs and markers of hypoxia in 3D ECs. RNA expression of the majority of the measured costimulatory markers was upregulated in cytokine treated 3D ECs relative to 2D or basal ECs. Expression of MHC class I and II as well as adhesion molecules ICAM and VCAM was also increased as a result of both 3D culture and cytokine treatment. Finally, in animal models of allotransplantation, ECs cultured in 2D caused a greater cytotoxic T cell response while ECs cultured in 3D resulted in greater T cell proliferation. ECs cultured in 2D also resulted in greater serum levels of IgM and IgG. Overall, these results show how culture condition, specifically substratum, determines the immune response to allotransplantation.

The key mechanisms that determine the long-term success of an organ transplant have been difficult to isolate because transplant outcomes are determined by the summation of a myriad of positive and negative signals. Engineered constructs made from biomaterials embedded with cells allow for unit dosing of cells in a controlled state which can be characterized at the time of implant, providing a method of investigating the relationship between phenotype and immune response under more precise conditions. However, they still allow for interaction between the host immune system and implanted cells. Thus, just as a biomaterial’s immunomodulatory properties can be adjusted and tested, the phenotypes and functional effects of matrix-adherent cells can be modified through changes to the substratum.

ECs are the ideal cell for studying the complex relationship between matrix-adherent cells and the immune system given their sidedness, alignment to flow, contiguity with neighboring cells, and need for adherence to a substratum. Matrix-adherence of ECs induces specific gene and protein expression patterns which affect physiology and immune recognition, enabling ECs to respond to a spectrum of perturbations. Past studies documented that 2D TCP substratums create greater mechanical stress on cells while 3D scaffolds made of natural materials, like collagen, enable energy states more in concert with what is seen *in vivo*. Though a wide range of scaffolds, cell types, and culture methods have been studied, the host immune response to cellularized scaffolds has not been well characterized, particularly in terms of the adaptive immune response.

### 4.1 RNAseq analysis

We determined that inflammatory states are substratum specific and that the addition of cytokines does not drive the 2D and 3D ECs towards a common phenotype (**Figure 1**). These finding highlight how phenotypic differences as a result of culture condition can alter functional EC response to cues after transplantation. In the context of organ transplantation, the effect of different treatment regimens may depend on the characteristics of the donor organ, not simply the host immune response.

We show that less mechanically stressed 3D ECs are more readily able to respond to external cues, such as cytokine stimulation, than are 2D ECs (**Figure 2**, **3**). This increased responsiveness may be essential to responding to inflammation in a way that promotes homeostasis. The data increasingly supports the concept that 2D culture on tissue culture polystyrene (TCP) instead constrains the cells, creating a dysfunctional 2D EC phenotype that may unresponsive to cytokine induced stress which ultimately pushes their response towards cell death. These changes occurred despite both 2D and 3D ECs reaching a state of non-proliferative confluence.

This work examined a range of different EC-immune signaling mechanisms. Those which differed the most between 2D and 3D ECs, and between basal and cytokine ECs are promising targets of future investigations. For example, changes in CD137, CD48, ICOSL, and CD80 expression were both substratum and cytokine specific and may be leading contributors to the differences observed in the *in vivo* studies of immune function (**Table 1**). In future studies, increasing the length of time of cytokine exposure may further elucidate the differential effects of 2D and 3D culture (29).

Currently, one of the main clinical metrics used to make decisions about the use of donor organs is ischemic time, yet this metric has limited predictive value (30, 31). If donor organs are able to be non-invasively characterized before transplantation, it may be possible to determine a relationship between the phenotype of implanted cells and response to different treatment regimens or patient outcomes. In the future, this would allow treatment to be more tailored rather than a one size fits all regime of immunosuppression.

Further, these results can also be applied to the novel systems of *ex vivo* organ perfusion which are currently being developed. This work provides a framework for understanding the relationship between *ex vivo* culture conditions and *in vivo* transplant outcomes. For example, we showed that the increased VCAM1, HVEM, and ICOSL gene expression observed in 3DSF ECs correlated with decreased acute and chronic immune cytotoxicity as well as acute IFNγ release and serum IgM (**Table 1**, **Figures 2**, **4**, **5**). Thus, *ex vivo* culture conditions that increase expression of these surface markers in ECs may result in better transplant outcomes, providing a way to evaluate organ suitability.

### 4.2 Allogeneic rat transplantation model

Earlier work in our laboratory showed that matrix-embedding affects intracellular signaling pathways, MHC class II expression, co-stimulatory and adhesion molecules which in turn influence immune response in transplantation (32–34). Our laboratory has shown that substratum modulus influences the immune reaction to implanted ECs (35). For example, matrix-embedded ECs drive a different balance of T-helper cells compared to ECs implanted in suspension (36). These cell-cell interactions make them particularly relevant for understanding immune response in transplantation.

Although previous studies in our lab have examined the role of ECs in xenogeneic transplants, here we chose to use an allogeneic transplant model in rats to study the role of substratum culture conditions. Allorejection is primarily mediated through direct antigen presentation while xenorejection is mainly indirect antigen presentation by recipient APCs (37). To examine the role of antigen presentation from the transplanted ECs, we performed transplants between two histoincompatible (major MHC mismatch) inbred rat strains, Brown Norway (RT-1^n^) and Lewis (RT-1^l^) rat, with Lewis rats as the recipients (38). Brown Norway (BN) to Lewis (LEW) transplants are commonly used in transplantation studies, particularly in models of lung and tracheal transplantation (39–42). The wealth of published surgical protocols for solid organ transplantation between these two species allows for further studies building upon the work outlined in this paper.

While the allogeneic model maximizes EC-immune cell interactions, there are differences in immune signaling between human and rat ECs. Vascular ECs in human allografts constitutively express MHC classes I and II (22, 43, 44). In comparison, previous studies have shown that rodents do not constitutively express MHC class II on all of their endothelium (3). T cell-EC co-stimulatory interactions pathways also vary between species, for example, CD2 signaling is more important to T cell activation in humans than in rodents (3). These and other differences of surface presentation between rodent and human ECs are potential limitations of our model. Efforts to individually target co-stimulatory molecules has been met with limited success, occasionally increasing transplant longevity in rodents but rarely translating to primates or humans (21). Additionally, by implanting functional cells (3D ECs) we aimed to affect a number of interconnected processes and in turn, yield a greater effect.

Previous investigations also showed how EC state differs with the addition of inflammatory cytokines. Presentation of foreign antigen, along with corresponding co-stimulatory signals, allows ECs to selectively regulate the migration of antigen-specific lymphocytes to locations of inflammation (45). ECs, however, do not express the same range of co-stimulatory molecules that are essential to signaling in professional APCs, limiting their ability to stimulate full T cell responses. Studies suggest that ECs can activate resting memory T cells to release a full range of cytokines (IL-2, IFNγ, IL-4) compared to bone-marrow derived APCs but are not able to stimulate naïve T cells to produce IFNγ at the same level without the addition of IL-12 (46).

### 4.3 Time scales of response *ex vivo*

We measured the immune response after *in vitro* co-culture at multiple time points ranging from 3 hours to 4 days. Early measurements of cytotoxicity and IFNγ release in the acute setting showed similar trends between the groups, with a greater response in all three experimental groups relative to the sham control, although only the 2Df SPs reached statistical significance (**Figure 4A**, **Supplementary Figure 9**).

In general, proliferation and cytotoxicity were inversely related. We postulate that the highly cytotoxic 2Df and 3Df SPs had a developed a memory response which allowed them to rapidly destroy the EC stimulus without causing a significant amount of proliferation. On the other hand, the 3DSF and sham SPs were the most proliferative since they were not as cytotoxic and thereby preserved the greatest number of cells to stimulate T cell proliferation (**Figure 4C**). This inverse relationship between cytotoxicity and proliferation was also observed in our assay with naïve allogeneic splenocytes which showed proliferation despite low cytotoxicity (**Supplementary Figures 10**, **11**). In this comparison, SPs from the 3DSF condition acted more similarly to naïve SPs than did splenocytes from either freed condition.

This *in vitro* response matches what likely occurs when ECs are implanted *in vivo*. Freed ECs are injected in suspension and are easily phagocytosed and cleared, removing the stimulus after a short time while the 3D ECs are adherent to the scaffold and retained for a longer period of time. Histology confirms that ECs remain in the scaffold for at least 5 days, allowing more opportunity to interact the host immune system (**Figure 7A**).

During organ transplantation, damage from surgery and ischemic reperfusion injury dislodges some donor ECs from their basement membrane. Thus, after transplant, the host immune system is likely to encounter ECs lining the blood vessels of the donor organ as well as damaged ECs circulating. This makes it particularly relevant to understand how immune cells interact with circulating ECs and if a greater number of circulating allogeneic cells could precipitate a greater allogeneic immune response.

### 4.4 Effects of physical barrier in matrix-adherent ECs

Histology of the *in vitro* cultured scaffolds further showed that the majority of the ECs were on the outer regions of the scaffold (**Figure 7A**). These regions were infiltrated with immune cells when the scaffolds were implanted *in vivo*. Staining of explanted scaffolds showed that, like ECs, immune cells did not reach the center of the scaffold, likely inhibited by the hypoxic environment at the center of the scaffold. They were able migrate approximately the same distance into the scaffold as did the d14 ECs and interact physically with the 3D ECs. Cytotoxicity was the lowest in 3DSF ECs in the chronically implanted animals and elevated in animals implanted with the easily accessible freed cells (**Figure 4A**). Longer term retention of the 3DSF ECs did not act as an adjuvant for cytotoxicity as might have been predicted.

Despite the limited cytotoxic effect observed for 3DSF implanted animals, the results of the booster experiment proved that 3DSF ECs can induce an *in vivo* memory response compared to sham animals. In the booster group, SPs from animals that originally received implants of 3DSF ECs in addition to the 2Df booster showed greater cytotoxicity and proliferation than the sham rats with only the booster injection (**Figure 4A, C**). This demonstrates that the immune cells are able to interact with the 3DSF ECs and develop a memory response *in vivo*, although that memory response differs from the one seen in animals implanted with freed ECs. In the acute setting, all the implanted ECs induced an immune response, however only 2DF ECs significantly increased responses associated with rejection, namely increased cytotoxicity and IFNγ release (**Figure 4A, B**). In the chronic setting, the immune cells of 3DSF implanted rats were not able to respond to and kill ECs *ex vivo* as quickly those implanted with freed ECs (**Figure 4C**).

Overall, this work contributes to a more complete understanding of how EC phenotype is determined by substratum and inflammation state and the importance of EC-immune cell interactions in determining transplant outcomes. Further work must to done to understand the specific effects of EC signaling on different T cell subsets. In addition, this 3D culture system could be used to evaluate the interactions of other non-immune antigen presenting cells such as epithelial and lymph node stromal cells.

## Data availability statement

The data presented in the study are deposited in the National Center for Biotechnology Information (NCBI) Sequence Read Archive (SRA) repository, accession number PRJNA838704.

## Ethics statement

The animal study was reviewed and approved by Massachusetts Institute of Technology Committee on Animal Care.

## Author contributions

The project was conceived by EW and EE. *In vitro* cell culture, RNAseq, and *in vivo* experiments were conducted, analyzed, and interpreted by EW. The paper was drafted by EW and revised by EE. All authors contributed to the article and approved the submitted version.

## Acknowledgments

We thank the Koch Institute’s Robert A. Swanson (1969) Biotechnology Center for technical support, specifically Flow Cytometry and Histology.

## Conflict of interest

The authors declare that the research was conducted in the absence of any commercial or financial relationships that could be construed as a potential conflict of interest.

## Publisher’s note

All claims expressed in this article are solely those of the authors and do not necessarily represent those of their affiliated organizations, or those of the publisher, the editors and the reviewers. Any product that may be evaluated in this article, or claim that may be made by its manufacturer, is not guaranteed or endorsed by the publisher.

## Supplementary material

Supplementary Figure 1 Sample-to-sample distances for 2D confluent and d3/d14 3DSF ECs. Sample-to-sample distances show how cytokine stimulation changes 2D and 3D EC gene expression. Differences between 2D basal and cytokine are slightly greater than those between 3D basal and cytokine.

Supplementary Figure 2 Heatmap of outcomes of TNF signaling pathway. Downstream effects from KEGG pathway of TNF signaling. TNF upregulated expression of different chemokines in 2D and 3D ECs. 3D ECs upregulated genes associated with cell adhesion, vascular effects, and cell survival compared to 2D ECs which upregulated genes associated with surface receptors and PRRs. Values are Z-scores.

Supplementary Figure 3 Heatmap of outcomes of IFNγ signaling pathway. Sorted by predicted function. Heatmap shows that IFNγ signaling leads to different downstream effects in 2D v 3D ECs. 3D ECs upregulate genes associated with vascular homeostasis and hematopoiesis while 2D ECs upregulated genes associated with cell cycle arrest and apoptosis. Value are Z-scores.

Supplementary Figure 4 Response to hypoxia and epithelial to mesenchymal transition in basal and cytokine stimulated ECs. 3D and 2D ECs maintained their distinct (A) hypoxia- and (B) mesenchymal transition-associated phenotypes with the addition of cytokines. Even with cytokine stimulation, 3D ECs were resistant to EndMT. **(A)** Sorted from average 3D EC value, high to low. **(B)** Sorted from average 2D EC value, high to low. Values are Z-scores. Heatmap of regulation of IFNγ genes. **(C)** Genes annotated “Regulation of IFNγ” within Response to IFNγ gene set (subset of GO:0034341). Both 2D and 3D ECs increased expression of genes associated with regulation of the pathway, showing that matrix-adherence did not prevent cytokines in the media from reaching the 3D ECs. Values are Z-scores.

Supplementary Figure 5 Schematic of T cell-EC receptor interactions. Effect of interactions between host T cells and donor endothelial cells. Green plus (+) denotes EC stimulation of T cells and red minus (-) denotes inhibition of T cell functions.

Supplementary Figure 6 Heatmap of genes associated with antigen process and presentation on MHC class I. Genes from Antigen Processing and Presentation GO code set (GO:0002474). 3D ECs showed greater upregulation of MHC class I (RT1-A) along with the associated antigen processing machinery, specifically Tap1 and Tap2. Values are Z-scores.

Supplementary Figure 7 Differentially expressed chemokines after cytokine stimulation. **(C, D)** Many chemokines were upregulated with the addition of cytokines, however 2D and 3D ECs increased gene expression of different subsets of chemokines. Chemokines differentially upregulated in 3D ECs were generally associated with adaptive immune cells such as T cells and DCs while those differentially upregulated in 2D ECs were associated with innate immune cells such as neutrophils and monocytes. Heatmap values are z-scores.

Supplementary Figure 8 Comparison of cytotoxicity, IFNγ Release, and proliferation in syngeneic (LEW) and allogeneic (BN) EC-SP co-cultures. Lewis (LEW) and Brown Norway (BN) SPs were co-cultured with BN ECs. **(A)** Co-culture with naïve SPs did not cause increased EC death in a 3 hour co-culture. **(B)** Allogeneic SPs trended towards greater IFNγ release compared to syngeneic SPs although the difference did not reach statistical significance. **(C)** Allogeneic SPs proliferated more in co-culture with ECs than alone while syngeneic SPs did not. **(D)** The proportion of the CD3+ cells within allogeneic co-cultures increased relative to allogeneic SPs alone and relative to syngeneic co-cultures. Statistics: two-way ANOVA.

Supplementary Figure 9 IFNγ release from SPs isolated from rats implanted with allogeneic ECs – 2 days. Although not statistically significant, all EC implanted groups trended towards greater IFNγ release than sham at 2 days. **(B)** Sham SPs showed significantly more IFNγ release than EC implanted groups between 2 and 4 days of *in vitro* culture. Compared to the sham group, implanted rats had a non-significant increase in IFNγ release at 2 days. Statistics: Ordinary one-way ANOVA. n=6.

Supplementary Figure 10 Proliferation in SPs isolated from rats implanted with allogeneic ECs – 2 days. MFI, mean fluorescence intensity. 3DSF SPs showed greater proliferation than 2Df and 3Df SPs in both the acute and chronic groups, after 2 days of co-culture. Nearly all proliferation appears to have occurred in the first two days of *in vitro* culture. Statistics: Ordinary one-way ANOVA. n=6.

Supplementary Figure 11 Proliferation of naïve allogeneic SPs co-cultured with ECs in different conditions. **(A)** SPs proliferated more after 4 days of co-culture in response to freed ECs compared to substratum-adherent ECs, whether adherent to 2D TCP or 3DSF. **(B)** Among the freed EC groups, were total cell number and target:effector ratio were constant, SPs proliferated more when co-cultured with 2D and d3 3D freed ECs than with d14 3D freed ECs. Statistics: Ordinary one-way ANOVA. n=3.
