# Supplementary figures and images for: Substratum interactions determine immune response to allogeneic transplants of endothelial cells
Source: Front Immunol. 2022 Aug 8;13:946794. doi: 10.3389/fimmu.2022.946794 (PMC9393654; doi:10.3389/fimmu.2022.946794)

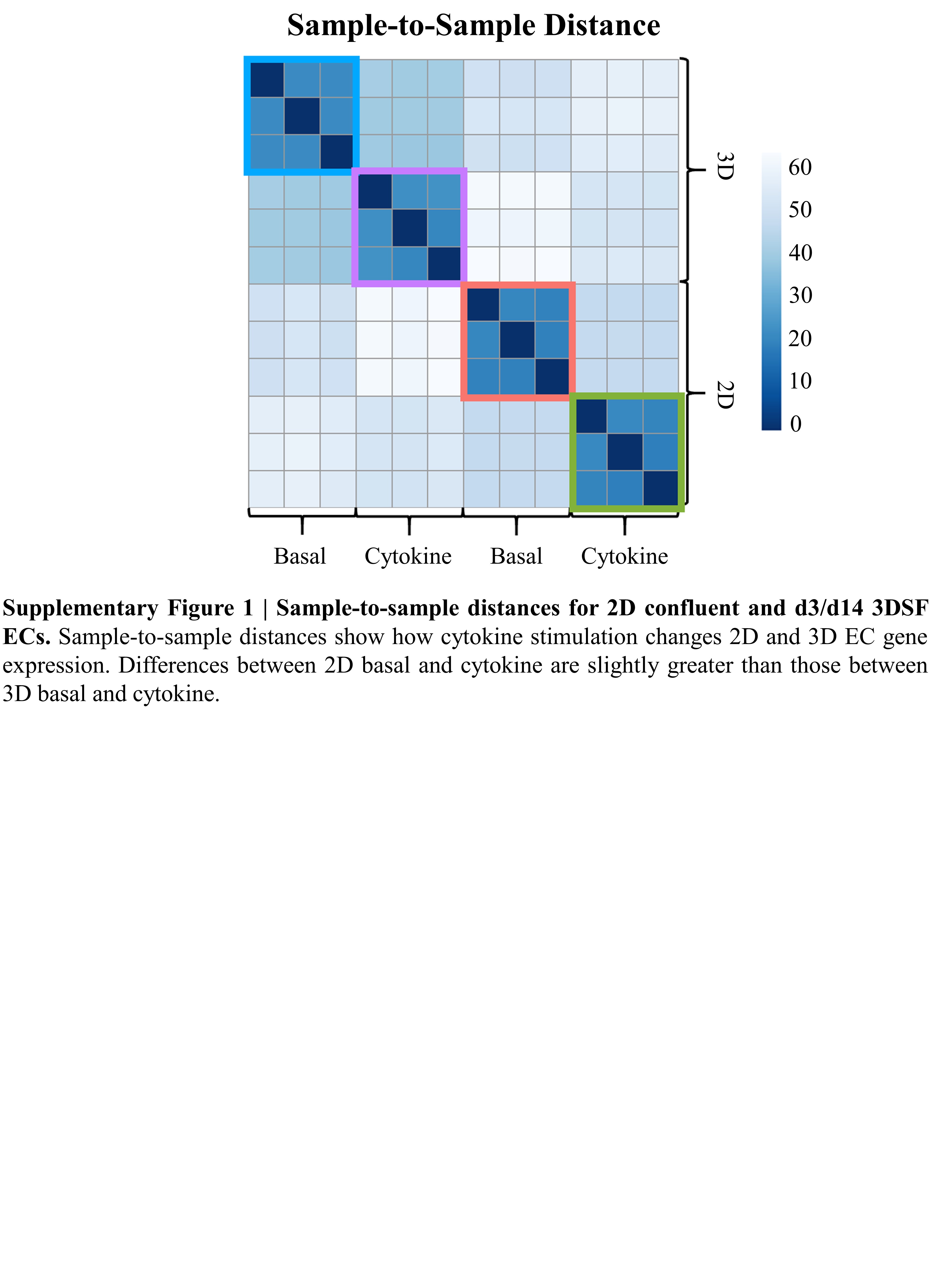

Supplement: Supplementary Figure 1 — Sample-to-sample distances for 2D confluent and d3/d14 3DSF ECs. Sample-to-sample distances show how cytokine stimulation changes 2D and 3D EC gene expression. Differences between 2D basal and cytokine are slightly greater than those between 3D basal and cytokine. [file Image_1.jpeg]

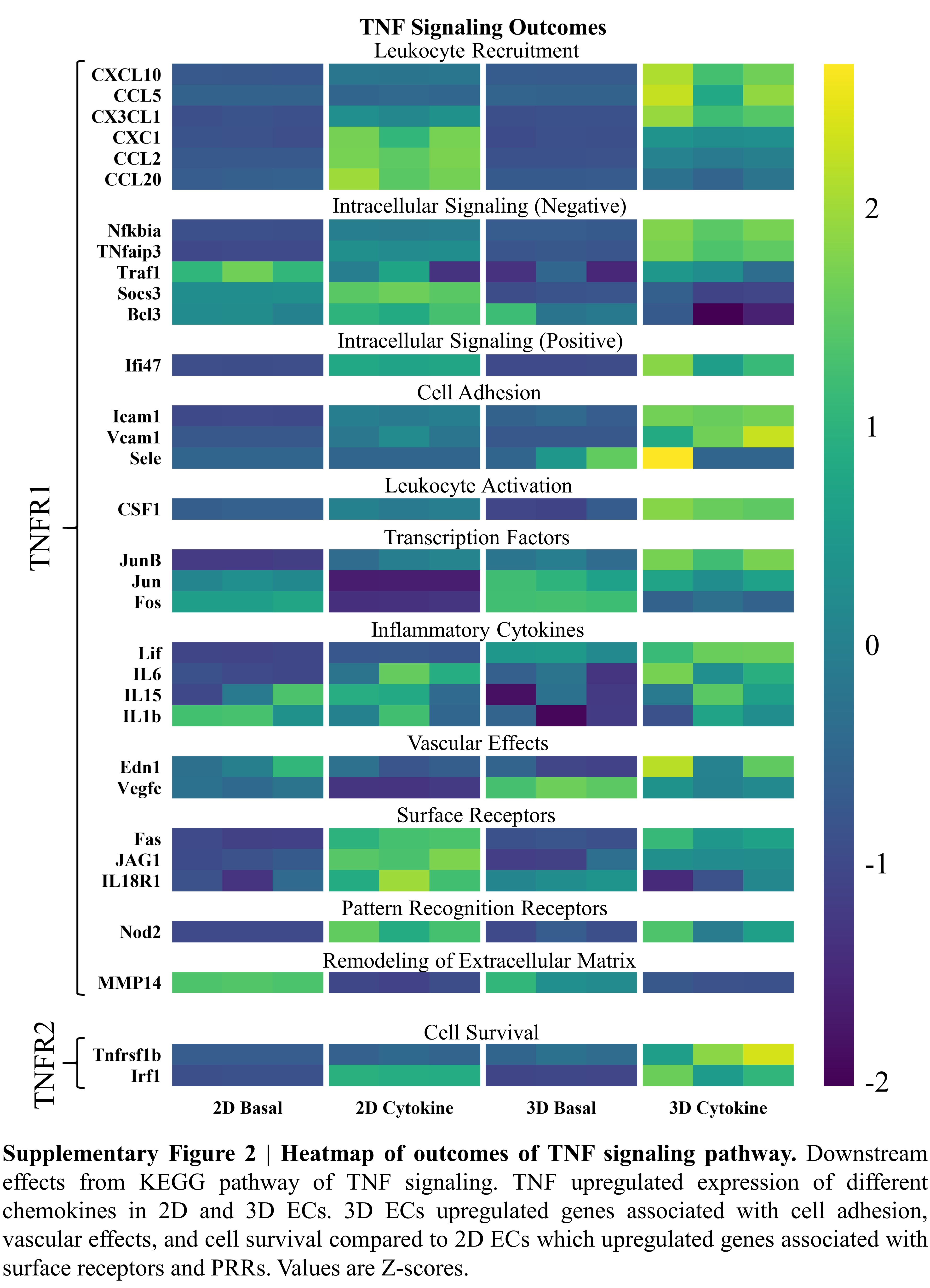

Supplement: Supplementary Figure 2 — Heatmap of outcomes of TNF signaling pathway. Downstream effects from KEGG pathway of TNF signaling. TNF upregulated expression of different chemokines in 2D and 3D ECs. 3D ECs upregulated genes associated with cell adhesion, vascular effects, and cell survival compared to 2D ECs which upregulated genes associated with surface receptors and PRRs. Values are Z-scores. [file Image_2.jpeg]

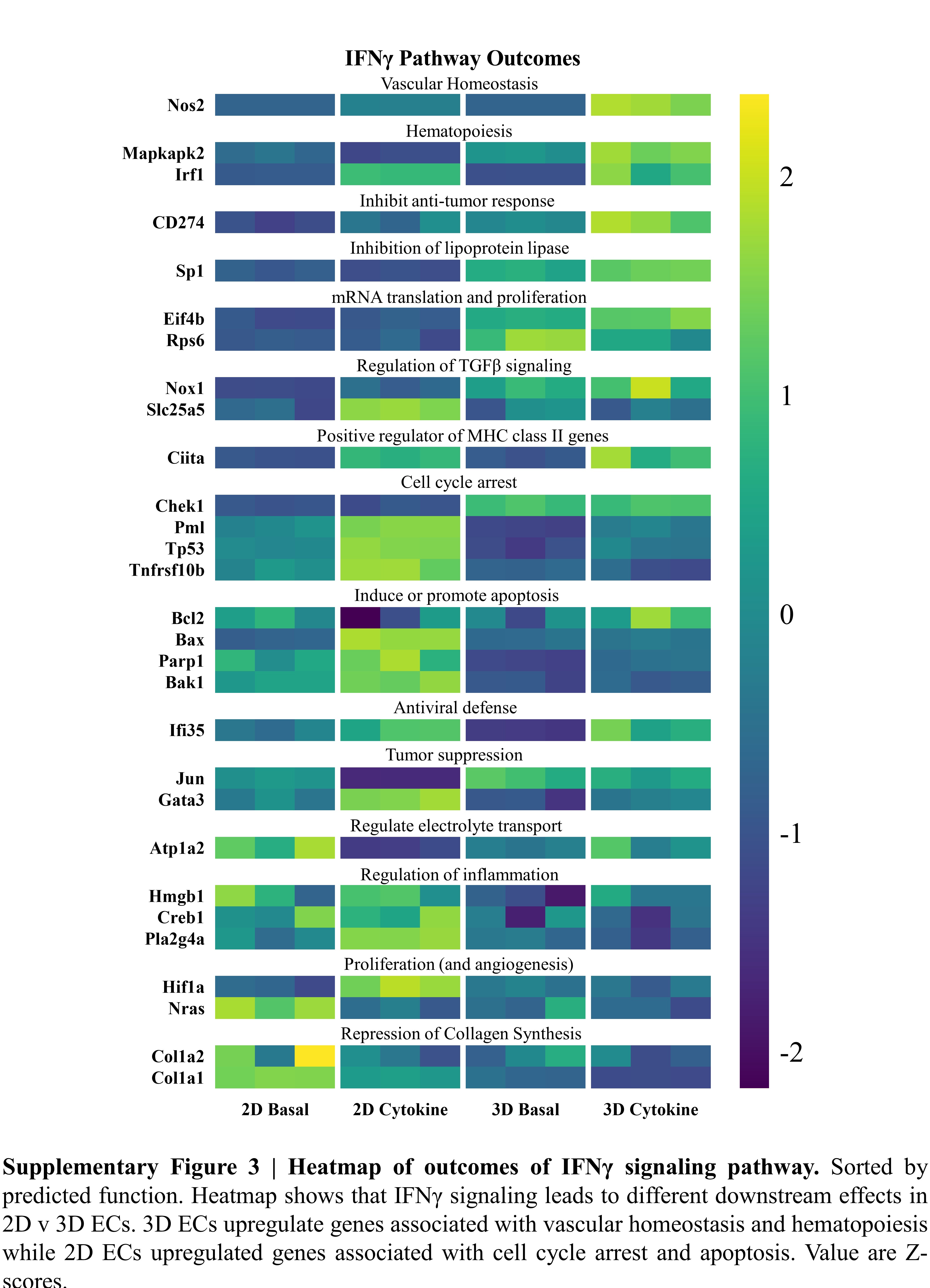

Supplement: Supplementary Figure 3 — Heatmap of outcomes of IFNγ signaling pathway. Sorted by predicted function. Heatmap shows that IFNγ signaling leads to different downstream effects in 2D v 3D ECs. 3D ECs upregulate genes associated with vascular homeostasis and hematopoiesis while 2D ECs upregulated genes associated with cell cycle arrest and apoptosis. Value are Z-scores. [file Image_3.jpeg]

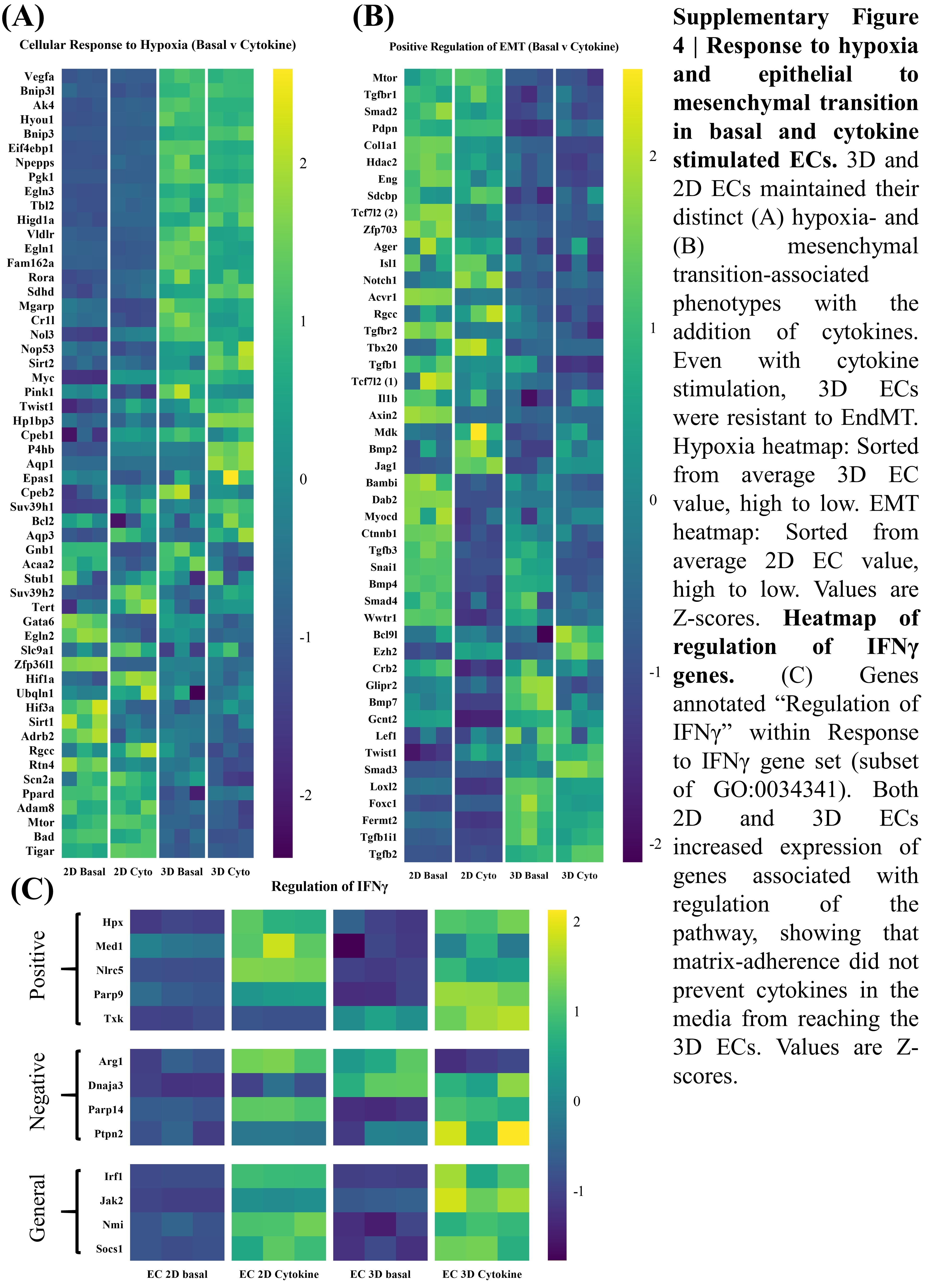

Supplement: Supplementary Figure 4 — Response to hypoxia and epithelial to mesenchymal transition in basal and cytokine stimulated ECs. 3D and 2D ECs maintained their distinct (A) hypoxia- and (B) mesenchymal transition-associated phenotypes with the addition of cytokines. Even with cytokine stimulation, 3D ECs were resistant to EndMT. (A) Sorted from average 3D EC value, high to low. (B) Sorted from average 2D EC value, high to low. Values are Z-scores. Heatmap of regulation of IFNγ genes. (C) Genes annotated “Regulation of IFNγ” within Response to IFNγ gene set (subset of GO:0034341). Both 2D and 3D ECs increased expression of genes associated with regulation of the pathway, showing that matrix-adherence did not prevent cytokines in the media from reaching the 3D ECs. Values are Z-scores. [file Image_4.jpeg]

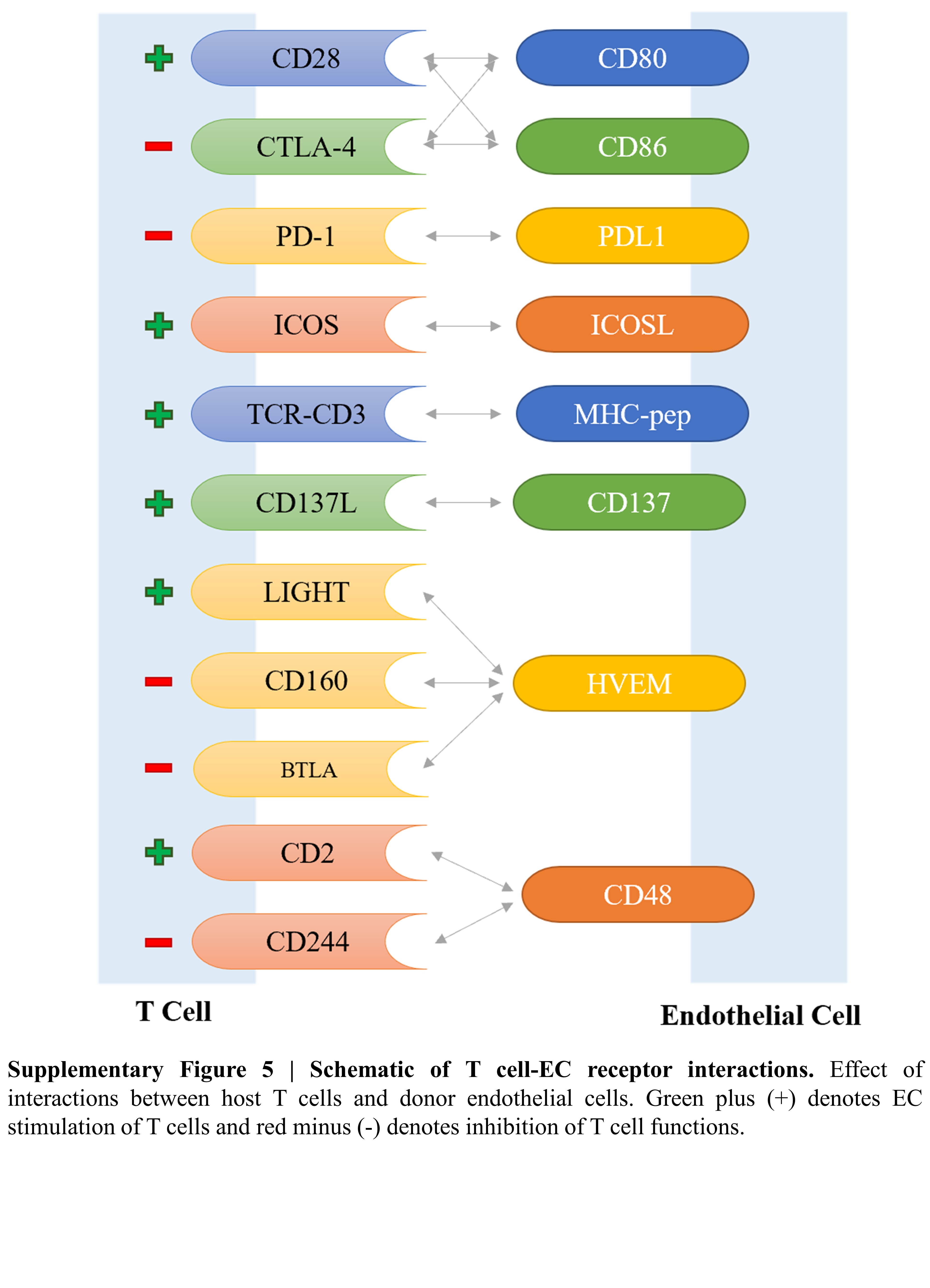

Supplement: Supplementary Figure 5 — Schematic of T cell-EC receptor interactions. Effect of interactions between host T cells and donor endothelial cells. Green plus (+) denotes EC stimulation of T cells and red minus (-) denotes inhibition of T cell functions. [file Image_5.jpeg]

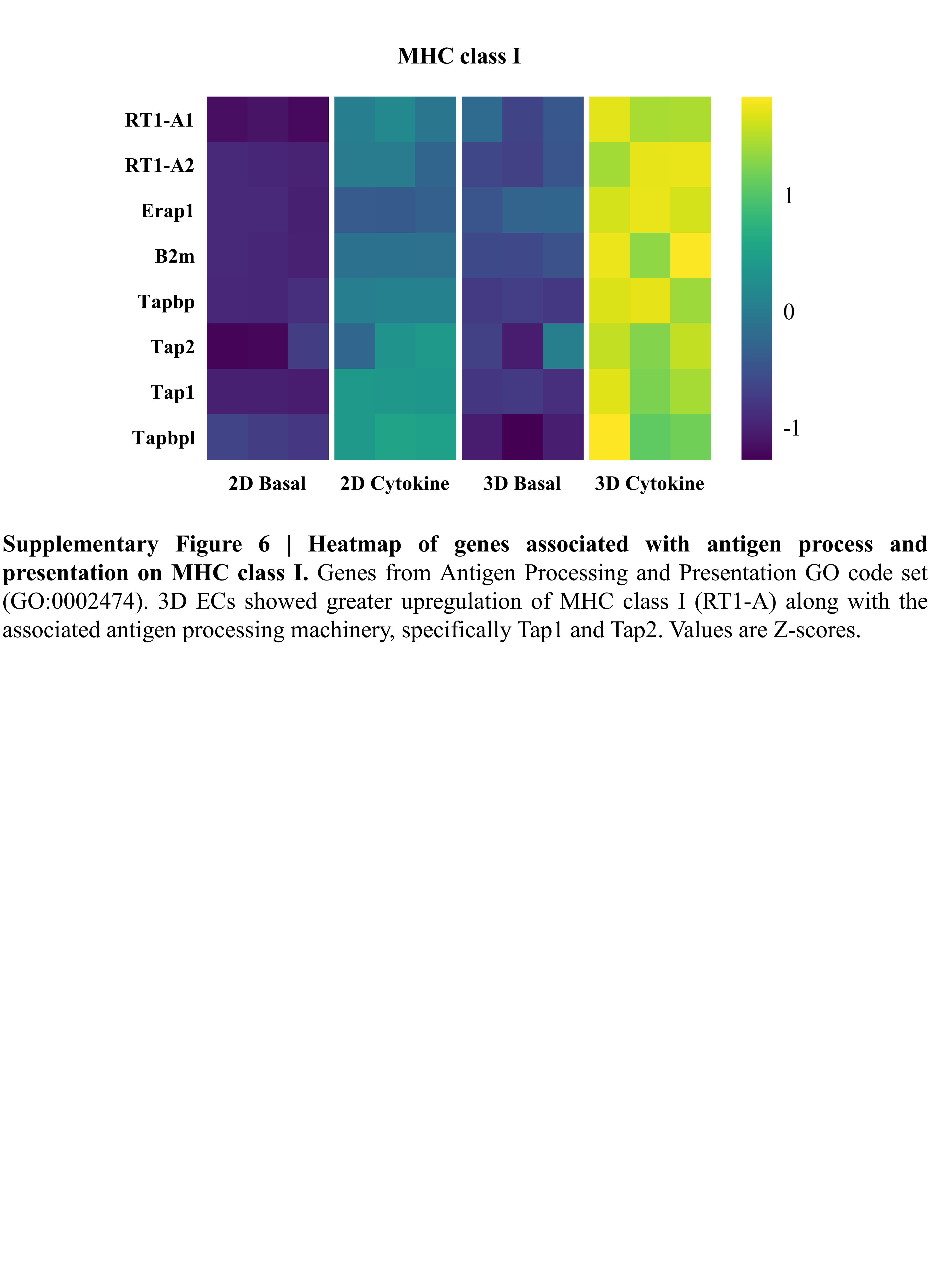

Supplement: Supplementary Figure 6 — Heatmap of genes associated with antigen process and presentation on MHC class I. Genes from Antigen Processing and Presentation GO code set (GO:0002474). 3D ECs showed greater upregulation of MHC class I (RT1-A) along with the associated antigen processing machinery, specifically Tap1 and Tap2. Values are Z-scores. [file Image_6.jpeg]

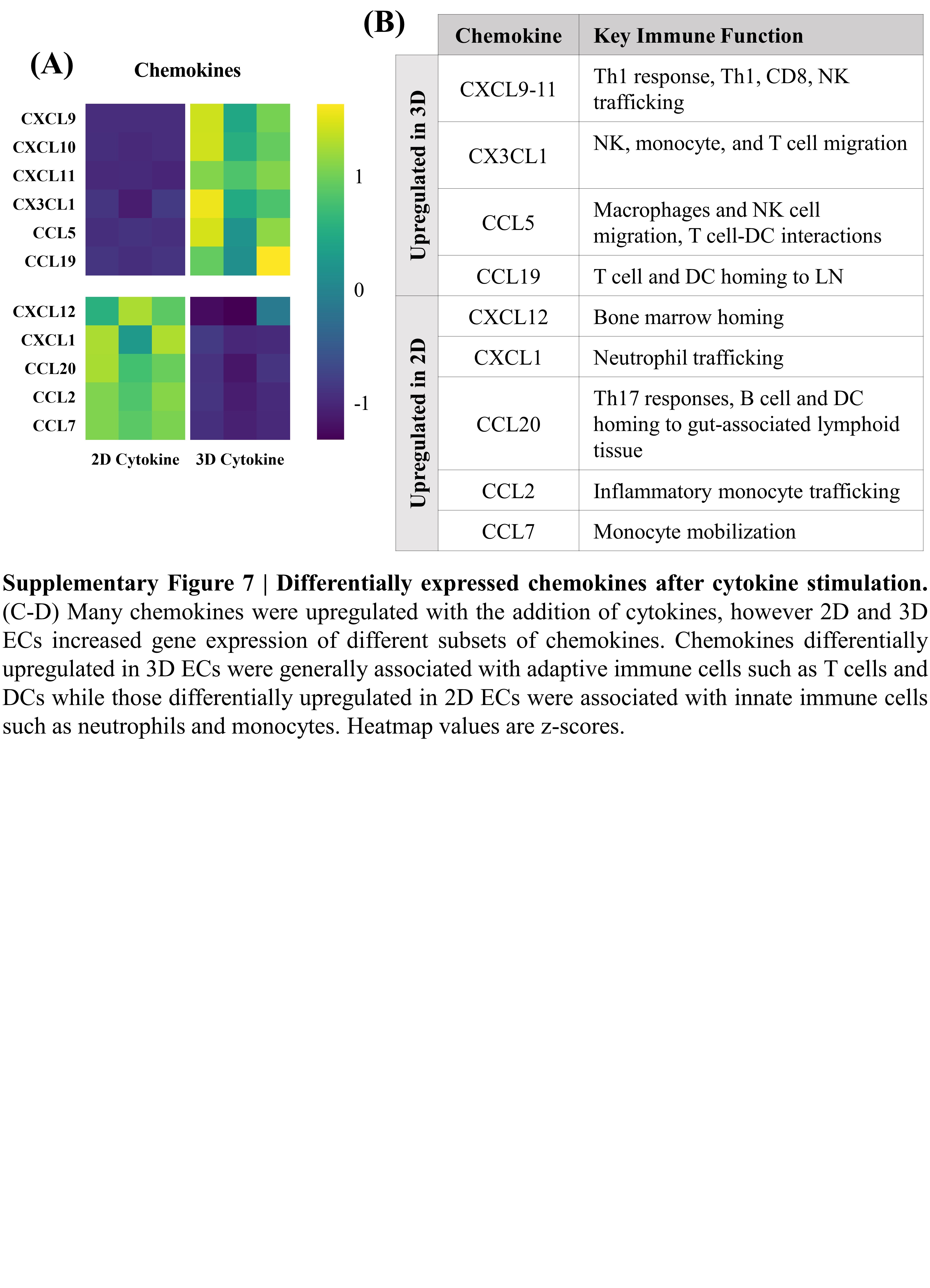

Supplement: Supplementary Figure 7 — Differentially expressed chemokines after cytokine stimulation. (C, D) Many chemokines were upregulated with the addition of cytokines, however 2D and 3D ECs increased gene expression of different subsets of chemokines. Chemokines differentially upregulated in 3D ECs were generally associated with adaptive immune cells such as T cells and DCs while those differentially upregulated in 2D ECs were associated with innate immune cells such as neutrophils and monocytes. Heatmap values are z-scores. [file Image_7.jpeg]

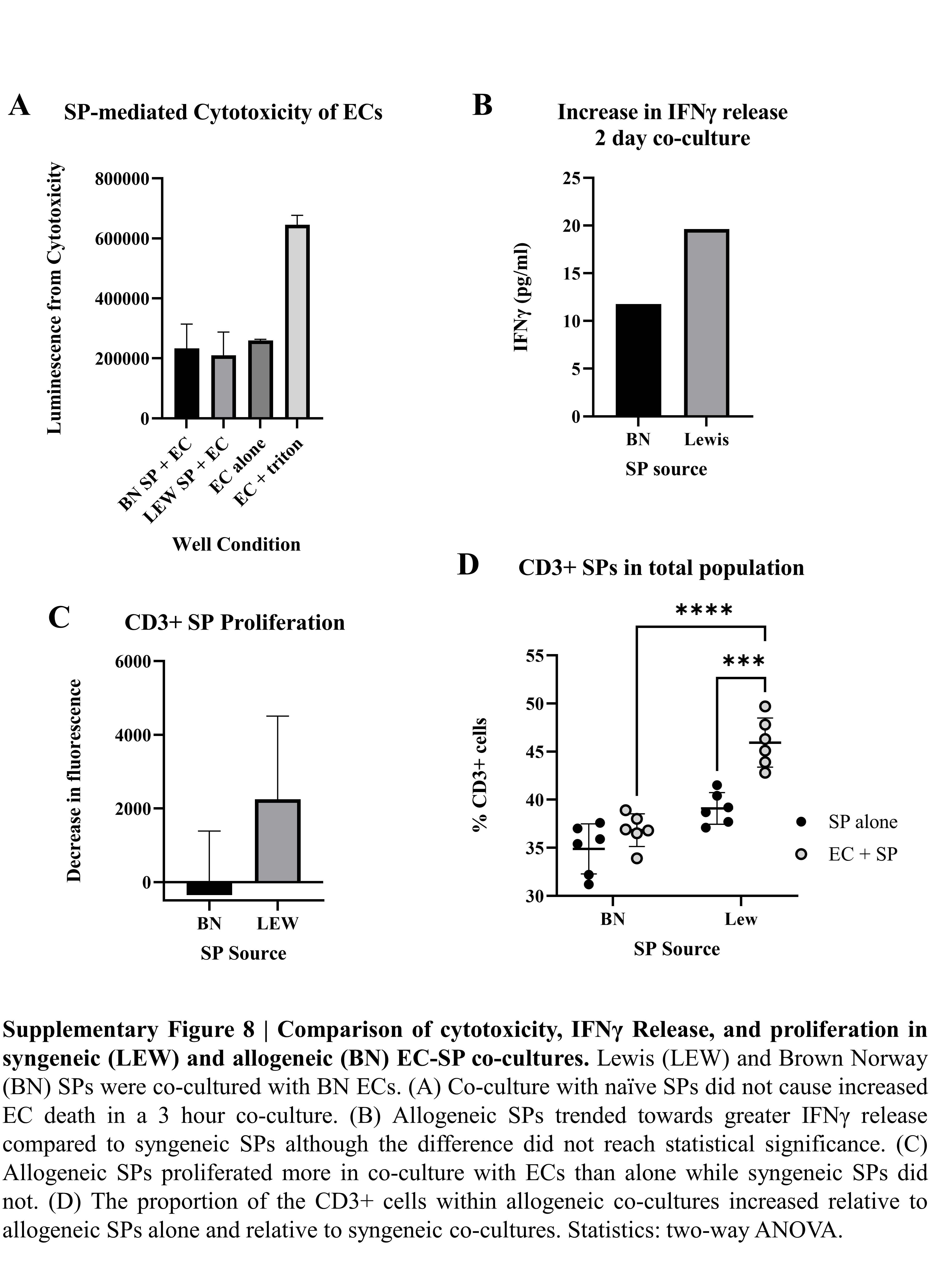

Supplement: Supplementary Figure 8 — Comparison of cytotoxicity, IFNγ Release, and proliferation in syngeneic (LEW) and allogeneic (BN) EC-SP co-cultures. Lewis (LEW) and Brown Norway (BN) SPs were co-cultured with BN ECs. (A) Co-culture with naïve SPs did not cause increased EC death in a 3 hour co-culture. (B) Allogeneic SPs trended towards greater IFNγ release compared to syngeneic SPs although the difference did not reach statistical significance. (C) Allogeneic SPs proliferated more in co-culture with ECs than alone while syngeneic SPs did not. (D) The proportion of the CD3+ cells within allogeneic co-cultures increased relative to allogeneic SPs alone and relative to syngeneic co-cultures. Statistics: two-way ANOVA. [file Image_8.jpeg]

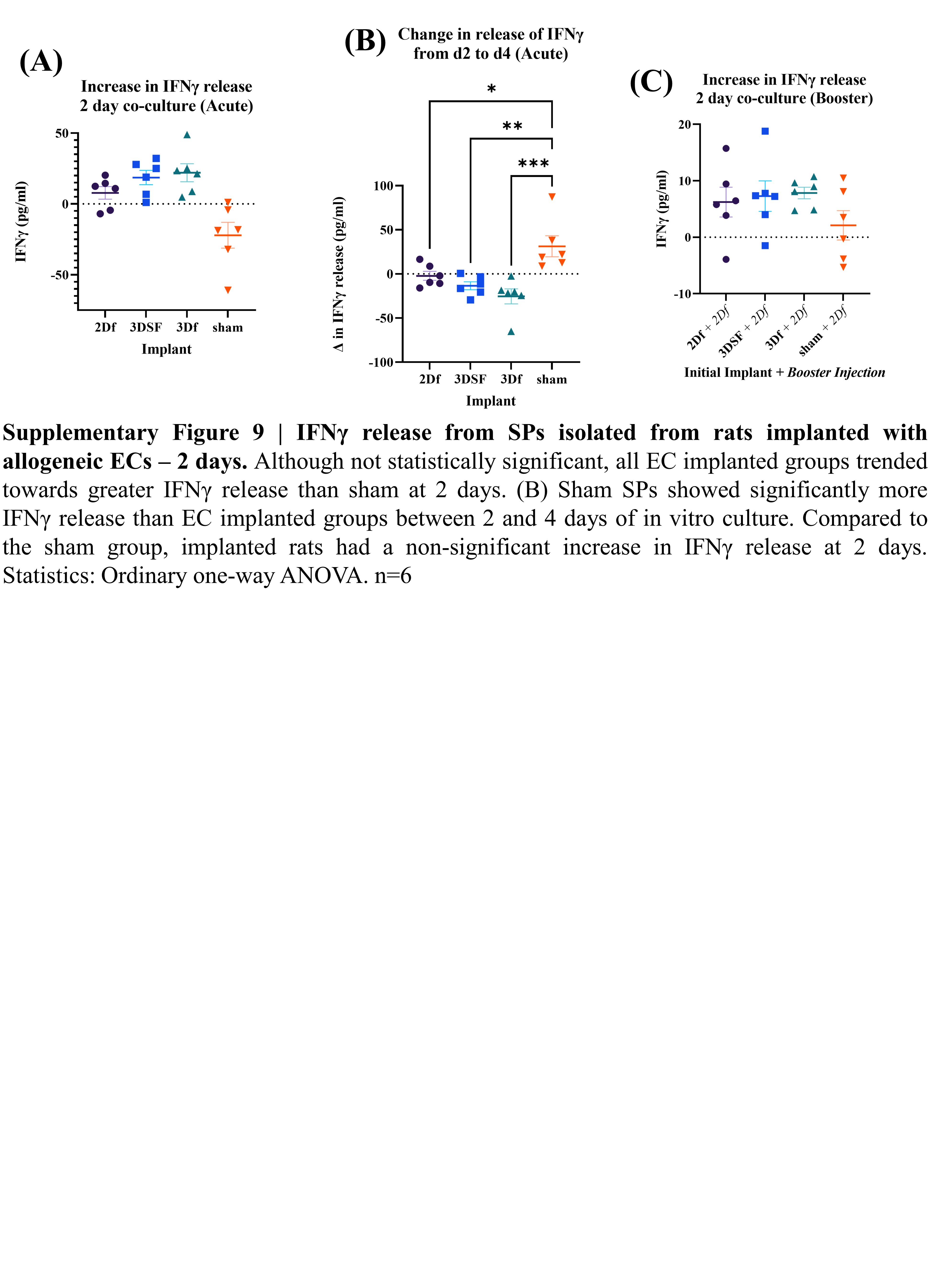

Supplement: Supplementary Figure 9 — IFNγ release from SPs isolated from rats implanted with allogeneic ECs – 2 days. Although not statistically significant, all EC implanted groups trended towards greater IFNγ release than sham at 2 days. (B) Sham SPs showed significantly more IFNγ release than EC implanted groups between 2 and 4 days of in vitro culture. Compared to the sham group, implanted rats had a non-significant increase in IFNγ release at 2 days. Statistics: Ordinary one-way ANOVA. n=6. [file Image_9.jpeg]

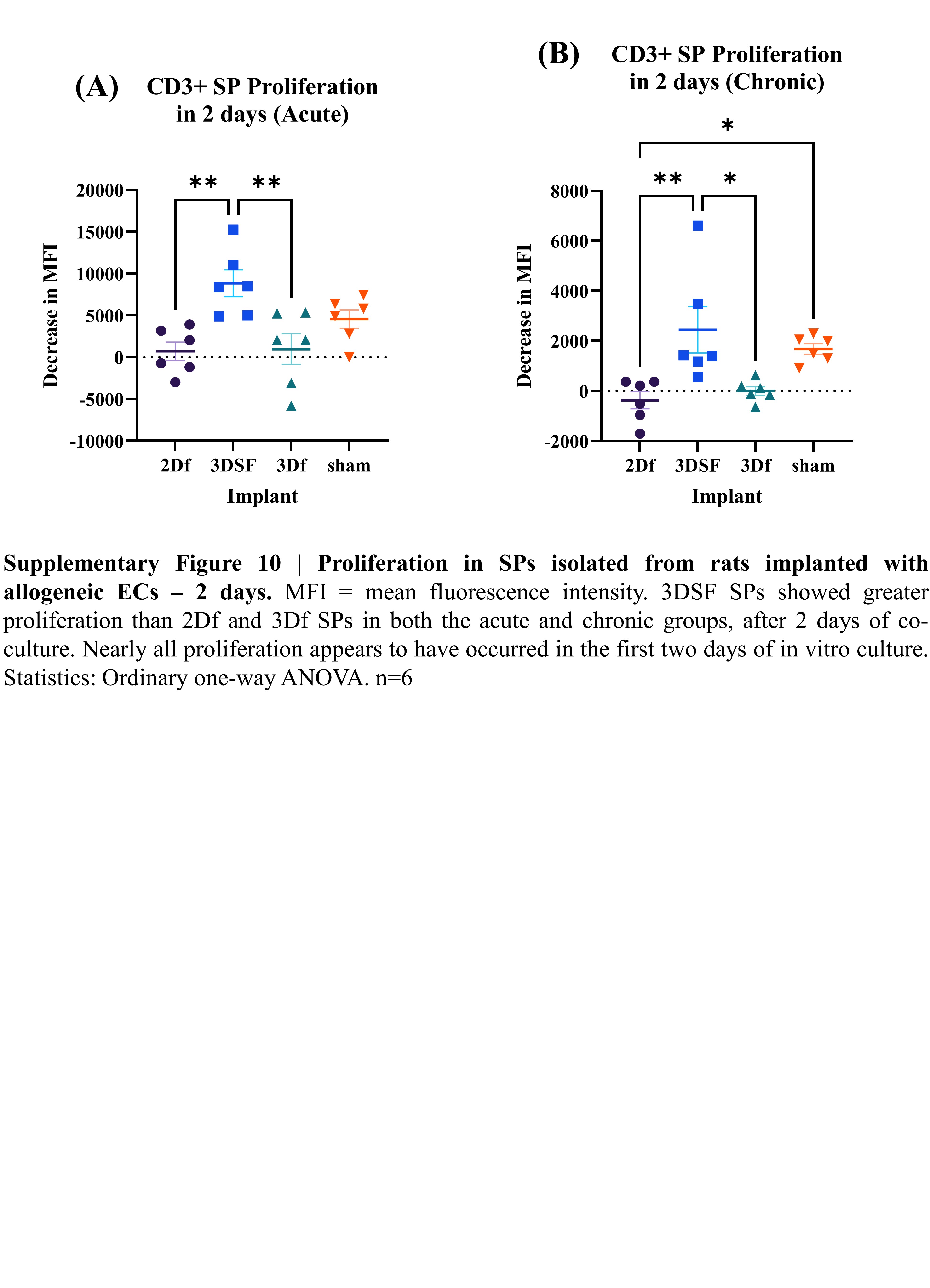

Supplement: Supplementary Figure 10 — Proliferation in SPs isolated from rats implanted with allogeneic ECs – 2 days. MFI, mean fluorescence intensity. 3DSF SPs showed greater proliferation than 2Df and 3Df SPs in both the acute and chronic groups, after 2 days of co-culture. Nearly all proliferation appears to have occurred in the first two days of in vitro culture. Statistics: Ordinary one-way ANOVA. n=6. [file Image_10.jpeg]

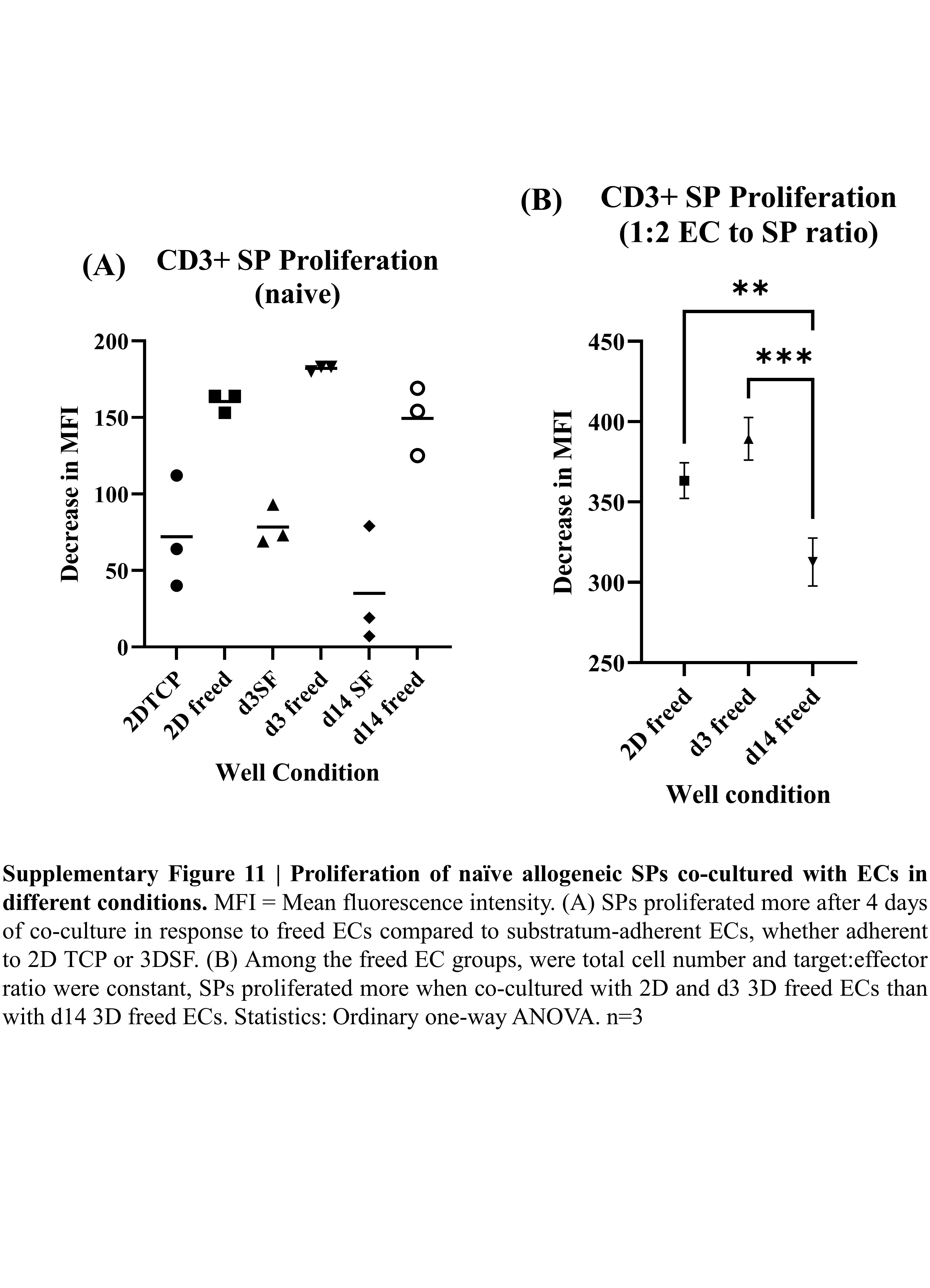

Supplement: Supplementary Figure 11 — Proliferation of naïve allogeneic SPs co-cultured with ECs in different conditions. (A) SPs proliferated more after 4 days of co-culture in response to freed ECs compared to substratum-adherent ECs, whether adherent to 2D TCP or 3DSF. (B) Among the freed EC groups, were total cell number and target:effector ratio were constant, SPs proliferated more when co-cultured with 2D and d3 3D freed ECs than with d14 3D freed ECs. Statistics: Ordinary one-way ANOVA. n=3. [file Image_11.jpeg]
